# PHKG2 confers resistance of ESCC to cisplatin and enhances CXCL8-dependent immunosuppression to exacerbate tumorigenesis

**DOI:** 10.1038/s41419-026-08721-1

**Published:** 2026-05-20

**Authors:** Jiayan Wu, Chengrui Zhong, Haosheng Zheng, Fei Qin, Xianyu Qin, Yuzhen Zheng, Junguo Chen, Gengfeng Wang, Bozhu Jian, Zui Liu, Xingping Yang, Chao Cheng, Guolin Li, Hongying Liao

**Affiliations:** 1 Department of Thoracic Surgery, The Sixth Affiliated Hospital, Sun Yat-sen University, Guangzhou, China; 2 Biomedical Innovation Center, The Sixth Affiliated Hospital, Sun Yat-sen University, Guangzhou, China; 3 Department of General Surgery (Hepatobiliary, Pancreatic and Splenic Surgery), The Sixth Affiliated Hospital, Sun Yat-sen University, Guangzhou, China; 4 University of Burgundy, Dijon, France; 5 Institute for Clinical and Translational Research, Baylor College of Medicine, Houston, TX USA; 6 Section of Epidemiology and Population Sciences, Department of Medicine, Baylor College of Medicine, Houston, TX USA; 7 Dan L Duncan Comprehensive Cancer Center, Baylor College of Medicine, Houston, TX USA

**Keywords:** Oesophageal cancer, Protein-protein interaction networks, Virtual screening, Phosphorylation, Transcriptomics

## Abstract

Cisplatin (DDP)-based chemotherapy serves as a foundational cornerstone in the clinical management of esophageal squamous cell carcinoma (ESCC). However, intrinsic or acquired resistance to DDP persists as a significant clinical challenge, severely undermining therapeutic efficacy and contributing to unfavorable patient prognoses. Through functional screening with a CRISPR/Cas9 knockout library, transcriptomic profiling of cisplatin-resistant cell lines and clinical specimens, and in vitro/in vivo validation assays, we identified PHKG2 as a key mediator of cisplatin resistance in ESCC. Mechanistically, PHKG2-mediated cisplatin resistance is driven by the phosphorylation of IGF2BP3 at residues T225 and T306. This post-translational modification enhances IGF2BP3 phase separation, thereby stabilizing CXCL8 mRNA in an m6A-dependent manner. Furthermore, increased CXCL8 secretion by ESCC cells induces the polarization of M0 macrophages toward an M2 phenotype within the tumor immune microenvironment, which subsequently suppresses the cytotoxicity of CD8⁺ T cells and fosters an immunosuppressive microenvironment in ESCC. Significantly, pharmacological inhibition of PHKG2 using prexasertib notably curtails ESCC cell proliferation and enhances cisplatin sensitivity. This study underscores the promising potential of targeting PHKG2 as a therapeutic approach to overcome cisplatin resistance in ESCC.

**Figure:**
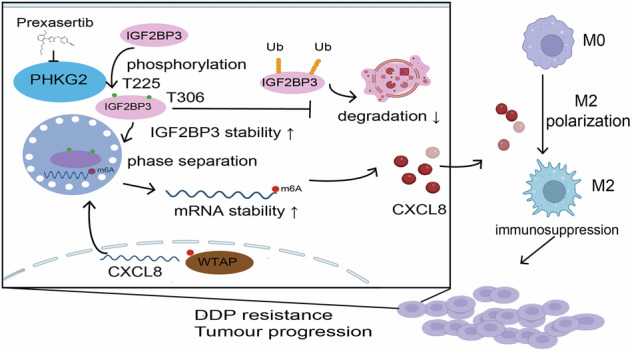

## Introduction

Esophageal cancer was ranked as the 11th most prevalent malignancy and the 7th leading cause of cancer-related mortality globally in 2022 [1, 2]. It comprises two major pathological subtypes, namely esophageal squamous cell carcinoma(ESCC) and esophageal adenocarcinoma, which differ in geographical distribution and molecular profiles [3]. ESCC is the predominant subtype, accounting for 85% of all esophageal cancer cases [2]. Chemotherapy, including neoadjuvant and adjuvant regimens, serves as a cornerstone of therapeutic strategy for ESCC patients [4, 5]. Among various chemotherapeutic approaches, cisplatin (DDP)-based neoadjuvant therapy has emerged as a promising intervention, significantly improving clinical outcomes in selected patients with locally advanced disease [6]. However, the clinical utility of DDP is often restricted by innate and acquired resistance [7]. Only 19–58% of ESCC patients receiving DDP-based neoadjuvant chemotherapy exhibit a therapeutic response [8]. while the response rate for those undergoing DDP-based palliative chemotherapy remains merely 35% to 45% [9]. The mechanisms underlying such drug resistance, which substantially undermine DDP’s clinical efficacy, have not been fully elucidated [10]. Thus, a more comprehensive understanding of the molecular basis of resistance to DDP-based chemotherapy may improve clinical outcomes of ESCC patients. Multiple biological processes, including metabolic reprogramming, apoptosis, DNA damage repair, and epigenetic modification, have been associated with DDP resistance [11]. However, the specific molecular mechanisms governing DDP resistance in ESCC remain unclear.

Protein phosphorylation plays a pivotal role in regulating diverse biological processes and is essential for signal transduction, modulation of protein activity, and governance of fundamental cellular events such as migration, proliferation, apoptosis, and differentiation [12, 13]. Accumulating evidence suggests that regulators of protein phosphorylation are involved in various cancers [14]. Targeting protein phosphorylase kinases has thus emerged as a highly promising therapeutic strategy to impede tumor progression and overcome therapeutic resistance [14, 15]. Phosphorylase kinase G2 (PHKG2) encodes the catalytic subunit of the phosphorylase kinase complex [16]. It belongs to the CaMK family [17]. and shares phosphorylation motifs with CaMKs and PKA [18]. Previous studies have demonstrated that PHKG2 phosphorylates peptides derived from the C-terminal domain of SIK3 [19]. Additionally, PHKG2 can upregulate intracellular iron levels, which are necessary for ferroptosis [20]. and has been significantly correlated with metabolic biological processes, patient survival, and therapeutic response [21]. However, the role of PHKG2 in DDP resistance in ESCC remains to be thoroughly investigated.

Insulin-like growth factor 2 mRNA-binding protein 3 (IGF2BP3) is a well-recognized oncofetal RNA-binding protein that plays a pivotal role in mediating chemoresistance [22]. As a key m⁶A reader, IGF2BP3 promotes the stability and translation of its target mRNAs in an m⁶A-dependent manner, thereby driving tumor progression and therapy resistance [23]. It contributes to chemoresistance in various cancers [24–26]. However, the specific role and underlying mechanisms of IGF2BP3 in chemoresistance in esophageal cancer remain poorly characterized and require further investigation.

In this study, we utilized genome-wide CRISPR/Cas9-based functional genomics approaches, along with unbiased transcriptomic profiling of clinical specimens and DDP-resistant cell lines, to systematically identify drivers of DDP resistance in ESCC. PHKG2, among the top candidates, was functionally validated as a key driver of DDP resistance in ESCC. Proteomic, transcriptomic, and epitranscriptomic approaches were employed to dissect the molecular mechanism by which PHKG2 mediates DDP resistance. IGF2BP3 was identified as a critical substrate of PHKG2, with phosphorylation at threonines 225 and 306 being essential for PHKG2-mediated DDP resistance. This modification promotes IGF2BP3 phase separation and thereby stabilizes CXCL8 mRNA in an m⁶A-dependent manner. Subsequently, elevated CXCL8 production in ESCC cells induces the polarization of M0 macrophages toward an M2 phenotype within the tumor immune microenvironment, suppresses the cytotoxicity of CD8⁺ T cells, and fosters an immunosuppressive ESCC microenvironment. In preclinical models, pharmacological inhibition of PHKG2 with the inhibitor prexasertib markedly suppressed ESCC proliferation and enhanced cisplatin sensitivity, providing insights for the development of novel combinatorial therapeutic strategies for ESCC.

## Materials and methods

### Cell lines and cell culture

Three human esophageal squamous cell carcinoma (ESCC) cell lines: KYSE-150, TE-1, and Eca109, were obtained from Wuhan Servicebio Technology Co., with certified short tandem repeat (STR) authentication. All the cell lines were contamination-free. Cells were cultured in Dulbecco’s Modified Eagle Medium (DMEM; Thermo Fisher Scientific) supplemented with 10% heat-inactivated fetal bovine serum (FBS; Gibco) under controlled conditions (37 °C, 5% CO₂).

### CCK-8, colony formation, invasion and apoptosis assays

For the CCK-8 assay, 1000 cells per well were seeded in 96-well plates and cultured with or without 1 μM DDP. Cell viability was monitored daily for 5 consecutive days using the CCK-8 reagent (Absin, abs50003). For the colony formation assay, 1000 cells were plated per well in 6-well plates, then cultured with or without 1 μM DDP for 14 days. The formed colonies were stained with crystal violet (MedChemExpress, HY-B0324A) and counted. For invasion assay, 4 × 10⁴ ESCC cells were resuspended in 300 µL serum-free DMEM and plated into the upper compartment of a 24-well plate. The lower compartment then received 600 µL DMEM mixed with 20% FBS (or specially treated conditioned medium). After 28 h of incubation at 37 °C, non-migratory cells in the upper layer were wiped off. Migratory cells were fixed with 4% paraformaldehyde for 15 min at room temperature and then stained with 0.1% crystal violet solution. Images were acquired using a light microscope, and quantification was done by counting cells in five randomly chosen fields per well. For the apoptosis assay, cells were treated with 30 μM DDP for 48 h, and then stained using the Annexin V-FITC/PI kit (Elabscience Biotechnology) according to the manufacturer’s instructions. The flow cytometry gating strategy is presented in Supplementary Fig. 8. Each experiment was conducted with three biological replicates.

### Immunoblotting (IB)

Protein extraction was performed by lysing cells with RIPA Lysis Buffer (Beyotime, P0013B). Total proteins were separated via SDS-PAGE electrophoresis and subsequently transferred to PVDF membranes (Millipore, Billerica). Membranes were blocked using 5% skim milk powder (Servicebio, China), then incubated sequentially with primary antibodies: anti-PHKG2 (Proteintech, 15109-1-AP, 1:1000), anti-IGF2BP3 (Proteintech, 14642-1-AP, 1:2000), anti-His (Proteintech, 66005-1-lg, 1:5000), anti-HA (Proteintech, 51064-2-AP, 1:5000), anti-CXCL8 (Proteintech, 17038-1-AP, 1:1000) and anti-GAPDH (Proteintech, 60004-1-Ig, 1:5000). Following PBS washing, the membranes were incubated with secondary antibodies: anti-mouse IgG, HRP-linked antibody (CST, 7076S, 1:2000) or anti-rabbit IgG, HRP-linked antibody (CST, 7074S, 1:2000) (Supplementary Table 1). Protein signals were visualized using an enhanced chemiluminescence detection kit (Meilunbio).

### Quantitative RT real-time PCR (qPCR)

Total RNA was extracted using RNA-Quick Purification Kit (ES Science, Guangzhou, China) and cDNA synthesis was performed using the color reverse transcriptions kit (EZBioscience, China) according to the manufacturer’s instructions. The reverse-transcribed cDNA products were used for qPCR analysis using FastSYBR PCR kit (CWBio, China). Supplementary Table 2 includes detailed information about the sequence of the used primers.

### siRNAs for PHKG2 and IGF2BP3

Small interfering RNAs (siRNAs) targeting PHKG2, IGF2BP3, and CXCL8 were procured from IGE Biotechnology (Guangzhou, China). Lipofectamine 3000 (Thermo Fisher Scientific, USA) was utilized for siRNA transfection. Twenty-four h post-transfection, the culture supernatant was replaced with fresh medium. Forty-eight h after cotransfection, qPCR and western blot analyses were conducted to assess the knockdown efficiency. The specific targeting sequences are detailed in Supplementary Table 3.

### Lentiviral production and infection

Lentiviral constructs encoding sgPHKG2, shCXCL8 and cDNAs for full-length PHKG2, IGF2BP3, and their mutants, were transfected using Lipofectamine 3000(Thermo Fisher Scientific, USA) into HEK293T following the manufacturer’s instructions(Supplementary Table 3). The supernatants containing lentivirus particles were harvested at 24 and 48 h after transfection and then were used to infect ESCC cells with polybrene (5.0 μg/ml, Beyotime). After 72 h of transduction, ESCC cells were selected with 1.0 μg/ml puromycin for 7 days.

### Genome-wide CRISPR cas9 knockout screen

**Fig. 1:**
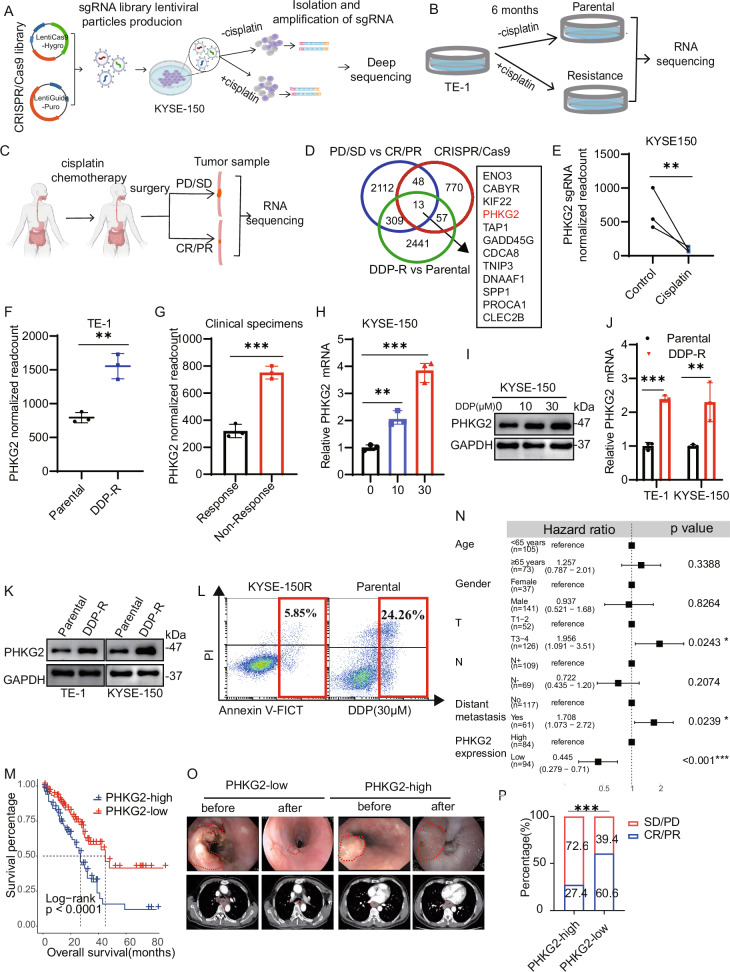
CRISPR screening and transcriptomic profiling of DDP-resistant cells and patient samples identify PHKG2 as a candidate driver of DDP resistance in ESCC. **A** Schematic workflows depict genome-wide CRISPR/Cas9 knockout library screening. **B** Schematic workflows depict DDP-resistant ESCC cell lines screening(*n* = 3). **C** Clinical sample analysis strategy comparing non-responders versus responders to DDP-based neoadjuvant therapy(*n* = 3). **D** Venn diagram identifies core candidate genes associated with DDP resistance through integrated CRISPR screening and RNA-seq of resistant cell lines and clinical samples. **E** PHKG2-targeting sgRNAs show significant decrease in DDP-treated versus control KYSE-150 cells(*n* = 3). **F** Elevated PHKG2 mRNA levels in DDP-resistant TE-1-R versus parental TE-1 cells(*n* = 3). **G** Increased PHKG2 mRNA expression in non-responsive (SD/PD) versus responsive (CR/PR) patient tumors(*n* = 3). **H** Dose-dependent upregulation of PHKG2 mRNA in KYSE-150 cells after 48 h DDP exposure (0, 10, 30 μM). **I** Corresponding PHKG2 protein elevation under identical DDP treatment conditions(*n* = 3). **J** Sustained PHKG2 mRNA overexpression in DDP-resistant KYSE-150-R and TE-1-R versus parental lines(*n* = 3). **K** Western blot confirmation of PHKG2 protein upregulation in resistant sublines. **L** Reduced DDP-induced apoptosis (30 μM, 48 h) in TE-1-R versus parental cells by Annexin V flow cytometry. **M** Kaplan-Meier survival curves were plotted to correlate PHKG2 expression with OS in ESCC. **N** Forest plot illustrating results from multivariate Cox regression analyses, depicting the prognostic performance of various variables as predictors of OS in ESCC patients. **O** Pre-and post-treatment computed tomography and endoscopic images of ESCC patients with low vs. high PHKG2 expression receiving DDP-based neoadjuvant therapy. **P** Distribution of therapeutic responses in the SYSU6H ESCC cohort evaluated by RECIST criteria after DDP-based neoadjuvant therapy.

In this study, a CRISPR human library (Ubigene, cat. no. UBISV230802JX3) was utilized to screen for genes contributing to DDP resistance in esophageal squamous cell carcinoma cells. The workflow of this forward genetic screening approach is depicted in Fig. 1A. To generate a stable Cas9-expressing ESCC cell line (KYSE-150-Cas9), lentivirus harboring the Cas9 coding sequence (Ubigene, cat. no. V-Cas-LV002-1000) was employed to infect KYSE-150 cells in the presence of polybrene (5.0 μg/ml, Beyotime). Post 72 h of transduction, the KYSE-150 cells underwent selection with 100 μg/ml Hygromycin (MCE, HY-B0490) for 7 days. Subsequently, the established KYSE-150-Cas9 cells were transduced with the CRISPR human library. Comprising 65,383 distinct single-guide RNA (sgRNA) sequences targeting 20,914 human genes, this transduction was executed at a low multiplicity of infection (MOI ~ 0.3) to ensure individual cell barcoding. The transduced cells were then selected with 1.0 μg/ml puromycin for 7 days, creating a mutant cell pool. This pool was treated with either vehicle or 1 μM DDP for 14 days. After treatment, a minimum of 5×10⁷ cells were harvested for genomic DNA extraction to achieve more than 400 fold coverage of the CRISPR human library. The sgRNA sequences were amplified with 2X PCR Master Mix, then subjected to high-throughput amplicon sequencing by EDITGene Technology (Guangzhou, China). Subsequently, the MAGeCK v0.5.7 algorithm was used to analyze sgRNA read counts.

### Immunoprecipitation, immunofluorescence and immunohistochemistry

For IP, cells were lysed by IP lysis buffer (Beyotime, P0013) supplemented with protease inhibitor cocktail (CWBIO, cw2383s), incubated on ice for 20 min, followed by centrifugation at 14,000 × *g* for 25 min at 4 °C. After pre-clearing with Protein A/G Magnetic Beads (MedChemExpress, HY-K0202), 2 mg of total protein lysate was used for immunoprecipitation. For immunofluorescence(IF), ESCC cells were plated in glass-bottom confocal dishes (Biosharp, BS-15-GJM). These cells were fixed with 4% paraformaldehyde. When analyzing ESCC tissues, Formalin-fixed paraffin-embedded tissues were employed. Both cell samples and tissue sections were permeabilized with 0.1% Triton X-100/PBS and blocked with goat serum(ZSGB Bio, ZLI9022). The primary antibodies used were anti-PHKG2 and anti-IGF2BP3. After being washed three times with PBS-T, the samples were incubated with Alexa Fluor 594-conjugated goat anti-rabbit IgG or Alexa Fluor 488-conjugated goat anti-mouse IgG. Subsequently, the samples were mounted with antifade mounting medium containing DAPI (Meilunbio), and imaging was performed using a ZEISS confocal microscope. For IHC, all human tissue research in this study adhered to the protocols approved by the Sixth Affiliated Hospital of Sun Yat-sen University, Guangzhou, China. The ESCC tissue sections were stained with the following antibodies: anti-PHKG2 antibody, anti-Ki67 antibody, and anti-cleaved-caspase 3 antibody, as detailed in Supplementary Table 1.

### RNA-Seq

Total RNA was extracted using Trizol and then utilized for RNA sequencing. Library preparation was carried out by Kindstar Sequenon Biotechnology. In brief, mRNA was isolated from the total RNA using poly-T oligo-attached magnetic beads. Fragmentation was achieved through the use of divalent cations at an elevated temperature in the NEBNext First Strand Synthesis Reaction Buffer (5X). First-strand cDNA synthesis was performed with a random hexamer primer and M-MuLV Reverse Transcriptase (RNaseH -). Subsequently, second-strand cDNA synthesis was carried out using DNA Polymerase I and RNase H. The remaining overhangs were converted into blunt ends via exonuclease and polymerase activities. After the adenylation of the 3’ ends of the DNA fragments, NEBNext Adaptors with a hairpin-loop structure were ligated to facilitate hybridization. To select cDNA fragments with a preferred length of 250-300 bp, the library fragments were purified using the AMPure XP system (Beckman Colter, Beverly, USA). Next, 3 μl of USER Enzyme (NEB, USA) was added to the size-selected, adaptor-ligated cDNA. The mixture was incubated at 37 °C for 15 min, followed by a 5-minute incubation at 95 °C before PCR. PCR was then performed using Phusion High-Fidelity DNA polymerase, Universal PCR primers, and Index (X) Primer. Finally, the PCR products were purified using the AMPure XP system, and the quality of the library was evaluated on the Agilent Bioanalyzer 2100 system. The clustering of the index-coded samples was executed on a cBot Cluster Generation System using the TruSeq PE Cluster Kit v3-cBot-HS (Illumina) following the manufacturer’s instructions. After cluster generation, the library preparations were sequenced on an Illumina Novaseq platform, generating 150-bp paired-end reads.

### MeRIP (m6A)-seq and RNA-immunoprecipitation-qPCR (RIP-qPCR)

For meRIP-seq, TRIzol™ Reagent (Invitrogen, 15596018) was used to extract total RNA from KYSE-150 cells. The Qubit RNA HS assay kit (Invitrogen, Q32852) was employed to measure the concentration of the total RNA. A quantity of 10 μg of the total RNA was fragmented into 100-200 nt RNA fragments with 10X RNA Fragmentation Buffer (composed of 100 mM Tris-HCl and 100 mM ZnCl₂ in nuclease-free H₂O). The reaction was halted by adding 10X EDTA (0.5 M EDTA). Methylated RNA immunoprecipitation was carried out using the EpiTM m6A immunoprecipitation kit (Epibiotek, R1804). Specifically, the fragmented RNA was first incubated with an anti-m6A monoclonal antibody (abcam, ab208577) at 4 °C for 3 h. Subsequently, protein A/G magnetic beads (Invitrogen, 8880210002D/10004D) were added, and the mixture was incubated at 4 °C for another 2 h to obtain immunoprecipitated RNA fragments. TRIzol™ Reagent (Invitrogen, 15596018) was then used to purify the m6A-enriched RNA. The library was prepared using the Smart-Seq method. Both the input samples that did not undergo immunoprecipitation (IP) and the m6A IP samples were sequenced on an Illumina NovaSeq 6000 sequencer with 150-bp, paired-end reads. For RIP-qPCR, cells were first collected. The cell pellet was then resuspended in lysis buffer and gently rotated at 4 °C for 30 min. After cell lysis, the lysate was obtained by centrifuging at 12,000 × *g* for 10 min. The supernatant was transferred to a new 1.5-ml tube. Approximately 10% of the lysate volume was set aside, and the RNA was extracted from this portion as the input for assessing RNA integrity. Subsequent RIP procedures were carried out using an RNA-immunoprecipitation kit (BersinBio, Bes5101). Forty microliters of protein G beads were washed twice with IP buffer. These washed beads, along with the antibody, were added to the lysate, and the mixture was incubated overnight at 4 °C. After the incubation, the supernatant was transferred to a fresh 1.5-ml tube. The beads were retrieved using a magnet and resuspended in 1× wash buffer. The suspension was rotated at 4 °C for 10 min. The supernatant was removed, and the washing step was repeated three more times. The co-precipitated RNA was extracted using the TRIzol Reagent and the Phenol-chloroform method. Both the co-precipitated RNA and the input RNA were used for cDNA synthesis with a color reverse transcription kit (EZBioscience, China) following the manufacturer’s instructions. The reverse-transcribed cDNA products were then used for qPCR analysis with a FastSYBR PCR kit (CWBio, China).

### Liquid chromatography mass spectrometry (LC-MS) analysis of PHKG2 interaction and IGF2BP3 phosphorylation

To identify the proteins that interact with PHKG2, the immune complexes were separated via SDS -PAGE and then stained using Coomassie brilliant blue (Servicebio, G2021). Regarding IGF2BP3 phosphorylation, IGF2BP3 was immunoprecipitated using an anti-IGF2BP3 antibody. Subsequently, the immune complexes were subjected to SDS -PAGE separation and stained with Coomassie brilliant blue (Servicebio, G2021). The peptides were then extracted and evaporated. Finally, they were sent to FitgeneBio for liquid chromatography mass spectrometry (LC-MS) analysis.

### Fluorescence recovery after photobleaching (FRAP) assay

The FRAP assay was conducted using a ZEISS LSM 880 confocal microscope equipped with Fast Airyscan and a 60× oil immersion objective. For the FRAP analysis of IGF2BP3 droplets, spots (1 mm in diameter) within 2 mm droplets were photobleached at 90% laser power with a 488 nm laser. Time-lapse images were captured post-photobleaching. Image analysis was performed using ZEISS software, while Prism software was utilized to generate and plot the FRAP curves.

### Dot blot assay

First, 200 ng of mRNA was isolated and denatured by heating for 3 min at 95 °C. Subsequently, the mRNA was diluted and spotted onto an Amersham Hybond-N+ membrane (LABSELECT, TM-NY-S-45), which is optimized for nucleic acid transfer. The membrane was then cross-linked under UV light. After that, the membrane was blocked with 5% non-fat milk in PBST for 1 h. It was then incubated with an anti-m6A antibody (Synaptic Systems, 202003, 1:2000) overnight at 4 °C. Next, the membrane was incubated with an anti-rabbit IgG (CST, 7074S, 1:3000) at room temperature for 1 h. The blots were detected using the ECL detection kit (Meilunbio). The same mRNAs were spotted on the membrane, stained with 0.02% methylene blue in 0.3 M sodium acetate (pH 5.2)(YuanYe, R28072) for 2 h and washed with RNase-free water for 30 mins.

### Patient‑derived tumor organoid (PDO) culture

Organoids were derived from esophageal squamous cell carcinoma patients with a tumor size of 0.5 cm3. The generation of organoids adhered to the manufacturer’s instructions (Baidi Biotech Ltd. 3D308-Kit), with the following detailed procedure: Fresh tissue samples were immediately transferred to specimen storage tubes containing an adequate amount of tissue transport preservation solution. Subsequently, the specimens were rinsed with DPBS-PS and digested using tissue digestion fluid. The isolated cells were then mixed with 5 μL of Matrigel (Corning, USA) and seeded into 96-well plates. Each well received 100 μL of supplemented culture medium, and the organoids were cultured in a humidified incubator at 37 °C with 5% CO₂.

### Organoid viability

Organoids were seeded into 96-well plates at 300–500 organoids in 5 μL of Matrigel per well in a total volume of 100 μL of the medium. Serially diluted compounds in 100 μL of medium were added to the cells 24 h later. After 4 days of incubation, Cell-Titer Glo Luminescent reagents (Promega, G7570) were added, and luminescence was measured. After 4 days of incubation, the medium was carefully aspirated, and 100 μL of live/dead reagents (Meilunbio, #MA0361-1) was added, followed by 20 min of incubation at room temperature. A fluorescence microscope was used to capture images of calcein AM (495 nm/515 nm) to represent the live cells, and PI (535 nm/617 nm) to identify the dead cells. The above assays were performed in triplicate.

### Cell‑derived tumor xenograft(CDX) mouse model

For the CDX tumor model, male BALB/c-nu mice with a body weight ranging from 20 to 23 grams were randomly divided into distinct groups. Subsequently, 1.0 × 10⁶ TE-1 or Eca109 cells were subcutaneously injected into the dorsal region of these nude mice. Once the tumors grew to approximately 50 mm³ in volume, the drug administration phase commenced. At this stage, the mice were re-randomized to receive treatments: either normal saline (NS) via intraperitoneal injection or 5 mg/kg of DDP administered intraperitoneally every three days. Approximately five weeks post-treatment, the mice were humanely sacrificed, and the tumor nodules were excised, collected, and photographed for further analysis.

### Patient‑derived tumor xenograft (PDX) mouse model

For the PDX tumor model, specimens were sourced from patients diagnosed with histologically confirmed esophageal squamous cell carcinoma (ESCC). PDX tumors were established by subcutaneously transplanting small tumor fragments, directly isolated from surgical specimens, into nude mice. The tumors were allowed to grow until reaching a diameter of 1.5 cm, at which point they were harvested. Ten percent of each harvested tumor was re-implanted into a new nude mouse, and this process was repeated for 3 to 4 generations before utilization in the experiment. The nude mice were randomly grouped and implanted with ESCC-PDX tumors. Once the tumors reached approximately 100 mm³ in size, the mice received either normal saline (NS) or DDP (DDP, 5 mg/kg) via intraperitoneal injection every 3 days. Additionally, intratumoral injections of either siNC or siPHKG2 (5 nmol per injection) were administered every three days. One month later, the mice were anesthetized, and the tumor volume and weight were subsequently measured.

### Statistics and analysis

The data are presented as mean ± SD. Statistical analysis was performed using GraphPad Prism(version 8.0.2) for Windows. Data were analyzed by one-way ANOVA, Student’s *t* test, or Chi-Square. Experiments were performed a minimum of three times. *P* < 0.05 was considered statistically significant. Other methods have been reported previously.

## Results

### CRISPR screening and transcriptomic profiling of DDP-resistant cells and patient samples identify PHKG2 as a candidate driver of DDP resistance in ESCC

To investigate the mechanisms underlying DDP resistance, we integrated genome-wide CRISPR screening with transcriptomic profiling of DDP-resistant cell lines and clinical specimens (Fig. 1A–C). The CRISPR screen was conducted in KYSE-150, an ESCC cell line with relatively low DDP sensitivity (IC₅₀ = 17.69 μM) compared to Eca109 (IC₅₀ = 5.75 μM) and TE-1 (IC₅₀ = 7.76 μM) (Supplementary Fig. 1A). A lentiviral CRISPR library targeting 21,914 human genes was transduced into Cas9-expressing KYSE-150 cells, which were then treated with 2 μM DDP or vehicle control for 14 days. Next-generation sequencing of genomic DNA identified 888 genes negatively associated with DDP resistance (log₂FC ≤ -1, *p* < 0.05) (Supplementary Fig. 1E and Supplementary Data 2). We established DDP-resistant sublines (TE-1-R and KYSE-150-R) through continuous exposure to DDP over 6 months (Fig. 1B). These resistant sublines exhibited significantly higher IC₅₀ values compared to their parental counterparts (Supplementary Fig. 1F). RNA sequencing analysis of TE-1-R versus parental TE-1 cells identified 1,654 upregulated genes (log₂FC ≥ 1, *p* < 0.05) (Supplementary Fig. 1G and Supplementary Data 3). Transcriptomic profiling of surgical specimens from advanced ESCC patients classified as either responders or non-responders to DDP-based neoadjuvant chemotherapy revealed 2247 genes upregulated in non-responders (log₂FC ≥ 1, *p* < 0.05) (Supplementary Fig. 1H and Supplementary Data 4).

Intersection analysis of these datasets identified 13 core resistance-associated genes, including PHKG2—a kinase previously linked to phosphorylation-dependent drug resistance (Fig. 1D) [14, 15]. CRISPR screening revealed a significant decrease of all three single guide RNAs targeting PHKG2 in DDP-treated KYSE-150 cells (Fig. 1E). Transcriptomic data corroborated the upregulation of PHKG2 in resistant sublines (TE-1-R vs. TE-1) and clinical non-responders (SD/PD vs. CR/PR) (Fig. 1F, G). DDP treatment induced the expression of PHKG2 mRNA and protein in KYSE-150 and TE-1 cells (Fig. 1H, I and Supplementary Fig. 1I, J), with sustained overexpression observed in resistant sublines (Fig. 1J, K). Notably, DDP-induced apoptosis was markedly reduced in resistant cells compared to parental controls (Fig. 1L and Supplementary Fig. 1K).

To investigate the clinical relevance of PHKG2 in ESCC, we assessed its differential expression in ESCC tissues via IHC (Supplementary Fig. 1L). IHC analysis of 178 ESCC tumor samples from patients receiving DDP-based neoadjuvant therapy showed that overall survival (OS) and disease-free survival (DFS) were significantly better in patients with low PHKG2 expression than in those with high PHKG2 levels (Fig. 1M and Supplementary Fig. 1M). Examination of the relationship between PHKG2 protein levels and clinicopathological characteristics revealed that high PHKG2 expression was associated with poor tumor differentiation, lymph node metastasis, and advanced clinical stage (Table 1). Multivariate Cox regression analysis confirmed PHKG2 expression as an independent prognostic factor for OS (Fig. 1N). Moreover, patients with favorable responses to DDP therapy exhibited lower PHKG2 expression than non-responders (Fig. 1O and Supplementary Fig. 1N). In the low-PHKG2 group, 60.6% (57/95) of patients achieved objective responses, compared with only 27.4% (23/84) in the high-PHKG2 group (Fig. 1P and Table 1). Collectively, these findings suggest that PHKG2 may confer resistance of ESCC to cisplatin treatment.

**Table 1:** Correlation between PHKG2 expression and clinicopathological features in patients with ESCC (*n* = 178).

Characteristic	PHKG2_High *n* = 84(%)	PHKG2_Low *n* = 94(%)	*p* value
Age			1
<65 years	49 (58.3%)	56 (59.6%)	
≥65 years	35 (41.7%)	38 (40.4%)	
Gender			0.7
Female	19 (22.6%)	18 (19.1%)	
Male	65 (77.4%)	76 (80.9%)	
T			1
T1-2	24 (28.6%)	28 (29.8%)	
T3-4	60 (71.4%)	66 (70.2%)	
N			0.8
N+	50 (59.5%)	59 (62.8%)	
N0	34 (40.5%)	35 (37.2%)	
Tumor differentiation			0.005
poor	73 (86.9%)	64 (68.1%)	
Well/Moderate	11 (13.1%)	30 (31.9%)	
Tumor location			0.5
Distal_third	35 (41.7%)	35 (37.2%)	
Middle_third	35 (41.7%)	47 (50.0%)	
Proximal_third	14 (16.7%)	12 (12.8%)	
Distant metastasis			0.4
No	52 (61.9%)	65 (69.1%)	
Yes	32 (38.1%)	29 (30.9%)	
Lymph node metstsis			< 0.001
(–)	27 (32.1%)	66 (70.2%)	
(+)	57 (67.9%)	28 (29.8%)	
Surgery			1
MIE	74 (88.1%)	84 (89.4%)	
no_MIE	10 (11.9%)	10 (10.6%)	
Complictions			0.04
No	75 (89.3%)	72 (76.6%)	
Yes	9 (10.7%)	22 (23.4%)	
Pathological response			0.03
CR/PR	23 (27.4%)	57 (60.6%)	
SD/PD	61 (72.6%)	37 (39.4%)	

*MIE* minimally invasive esophagectomy.

### PHKG2 drives DDP resistance in vitro and in vivo

**Fig. 2:**
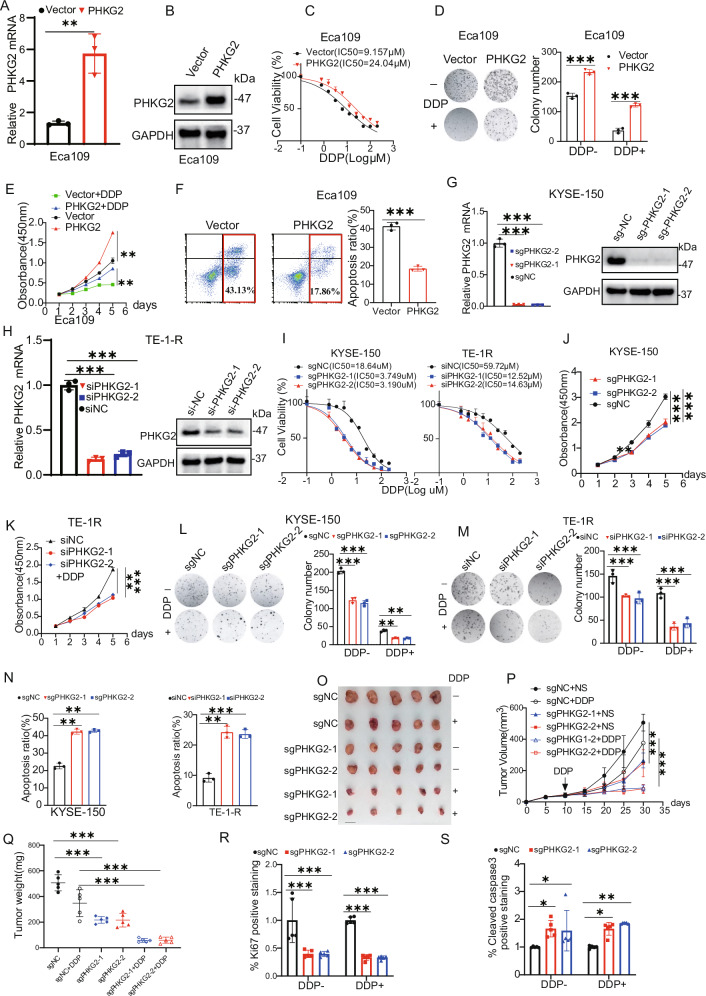
PHKG2 drives DDP resistance in vitro and in vivo. **A** qPCR analysis of PHKG2 mRNA levels in PHKG2 OE versus control Eca109 cells(*n* = 3). **B** Western blot analysis of PHKG2 protein OE in Eca109 cells. **C** The IC50 of DDP in PHKG2 OE and control Eca109 cells(*n* = 3). **D**–**F** The effects of PHKG2 OE on cell growth (0.5 μM DDP) as shown by CCK-8 assay, colony formation, and on apoptosis (30 μM DDP) by flow cytometry analysis(*n* = 3). PHKG2 KO or KD efficiency confirmed by qPCR (**G**) and western blot (**H**). **I** The IC50 of DDP in PHKG2 KO/KD and control cells(*n* = 3). **J**–**M** PHKG2 KO/KD potentiates DDP-mediated growth suppression (0.5 μM DDP, CCK-8/colony formation assays, *n* = 3). **N** Increased DDP-induced apoptosis (30 μM DDP) in PHKG2-KO/KD cells by Annexin V flow cytometry(*n* = 3). PHKG2 KO sensitizes TE-1 xenografts to DDP (5 mg/kg): tumors image (**O**), growth curves (**P**), and tumor weights (**Q**) in nude mice (*n* = 5). Scale bars: 1 cm. **R**, **S** IHC analysis of Ki67 and cleaved caspase 3 in TE-1 xenografts treated with or without DDP (5 mg/kg).

To investigate the functional role of PHKG2 in DDP resistance, PHKG2 was overexpressed (PHKG2 OE) in Eca109 cells (Fig. 2A, B). PHKG2 OE significantly increased the IC₅₀ of DDP in Eca109 cells compared with vector controls (Fig. 2C). Notably, PHKG2 OE not only enhanced cell growth under DDP treatment but also promoted proliferation in the absence of DDP (Fig. 2D, E), suggesting its dual functions in tumor progression and chemoresistance. Additionally, PHKG2 OE markedly attenuated DDP-induced apoptosis (Fig. 2F).

Subsequently, the impact of PHKG2 knockout(KO) or knockdown(KD) was evaluated in KYSE-150, TE-1, and TE-1-R cells (Supplementary Fig. 1A). Two independent sgRNAs achieved efficient PHKG2 knockout, as confirmed by qPCR and western blot (Fig. 2G and Supplementary Fig. 1A). Similarly, two distinct siRNAs effectively downregulated PHKG2 expression (Fig. 2H). PHKG2 KO or KD decreased the IC₅₀ of DDP in KYSE-150 and TE-1-R cells (Fig. 2I) and enhanced DDP-mediated growth inhibition in colony formation and Cell Counting Kit-8 (CCK-8) viability assays (Fig. 2J–M and Supplementary Fig. 2C, D). Flow cytometry analysis of Annexin V staining further indicated increased DDP-induced apoptosis in PHKG2 KO or KD cells compared with controls (Fig. 2N and Supplementary Fig. 2E, F).

For in vivo validation, mice bearing tumors derived from PHKG2 KO or control TE-1 cells were treated with DDP or vehicle (Supplementary Fig. 2G). PHKG2 KO sensitized tumors to DDP, as evidenced by smaller tumor volumes, lower tumor weights, decreased Ki67 expression, and increased cleaved caspase-3 levels compared with controls (Fig. 2O–S and Supplementary Fig. 2H). Collectively, these results demonstrate that PHKG2 promotes ESCC cell proliferation and DDP resistance in vitro and in vivo.

### PHKG2 stabilizes IGF2BP3 protein through phosphorylation of IGF3BP3 at T225 and T306

**Fig. 3:**
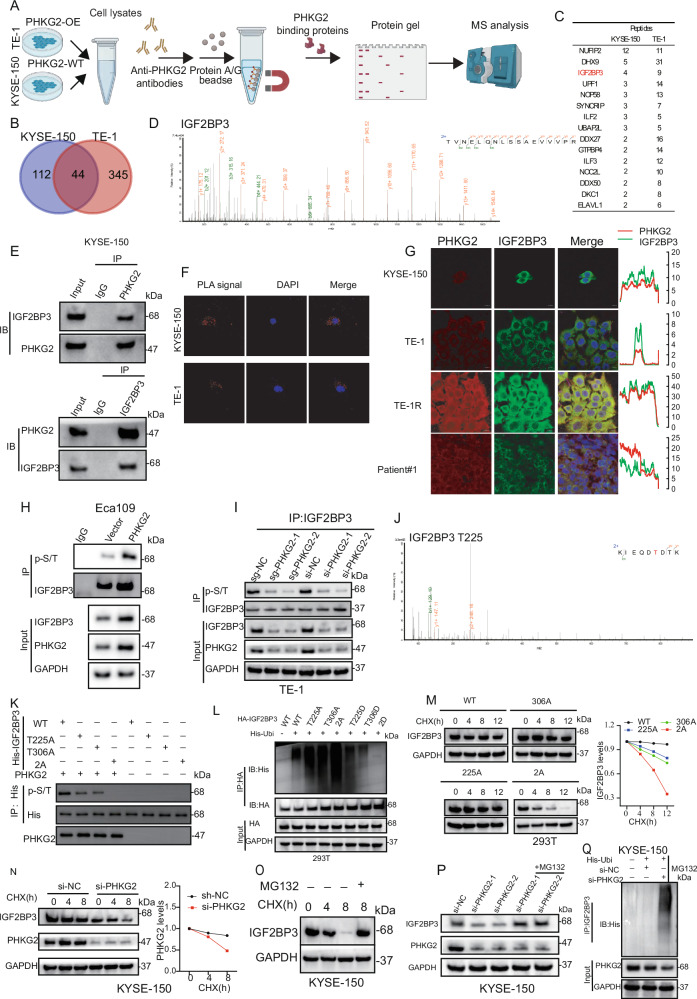
PHKG2 stabilizes IGF2BP3 protein through phosphorylation of IGF3BP3 at T225 and T306. **A** Co-immunoprecipitation assay was conducted in ESCC cells to examine protein-protein interactions. **B** A Venn diagram illustrated 44 common proteins isolated by PHKG2 immunoprecipitation in KYSE-150 and TE-1 cells, analyzed via liquid chromatography-tandem mass spectrometry (LC-MS/MS). **C** The quantity of peptide fragments from PHKG2-associated proteins in KYSE-150 and TE-1 cells was determined through IP-MS analysis. **D** A fragmentation spectrum of IGF2BP3 peptides identified by LC-MS/MS provided structural evidence of the interaction. **E** Co-immunoprecipitation followed by western blot analysis confirmed endogenous interaction between PHKG2 and IGF2BP3 in KYSE-150 cells. **F** PLA with anti-PHKG2 and IGF2BP3 antibodies in TE-1 and KYSE-150 cells. **G** Immunofluorescence staining demonstrated co-localization of PHKG2 and IGF2BP3 in ESCC cell lines and patient-derived tumor specimens. **H** Western blot analysis of immunoprecipitated IGF2BP3, using a phosphorylation-specific antibody, showed that PHKG2 overexpression enhanced IGF2BP3 phosphorylation in Eca109 cells. **I** Phosphorylation levels of immunoprecipitated IGF2BP3 were assessed via western blotting in TE-1 cells with PHKG2 KO or KD, revealing reduced phosphorylation. **J** Liquid chromatography-tandem mass spectrometry (LC-MS/MS) analysis identified the fragmentation spectrum of the IGF2BP3 T225 peptide. **K** In vitro kinase assays for p-S/T of IGF2BP3 in HEK293T cells co-expressed HA-PHKG2 with His-IGF2BP3 WT, S11A, T21A or 2 A mutant. **L** Western blotting indicated that single T225A/T306A mutations had minimal impact on protein stability, whereas the 2 A mutant exhibited significantly increased ubiquitylation compared to WT. In contrast, the phosphorylation mimic 2D mutant (T225D/T306D) showed reduced ubiquitylation compared to WT. **M** Western blot analysis revealed accelerated protein degradation in the 2 A mutant compared to IGF2BP3 WT. **N** Reduced IGF2BP3 protein levels were detected by western blotting in KYSE-150 cells with PHKG2 knockout versus control cells. **O** Western blotting showed that proteasome inhibitor MG132 treatment rescued IGF2BP3 degradation in KYSE-150 cells. **P** Western blot analysis demonstrated that MG132 treatment restored IGF2BP3 protein levels in PHKG2 KD KYSE-150 cells. **Q** Western blotting analysis of immunoprecipitated IGF2BP3 to determine the effect of PHKG2 KD on ubiquitylation of IGF2BP3 in KYSE-150 cells using.

Given that PHKG2 exerts its functions via its phosphorylase kinase activity, liquid chromatography-mass spectrometry(LC-MS) analysis was performed to profile the PHKG2-associated proteins in KYSE-150 and TE-1 cells for substrate identification (Fig. 3A). Forty-four PHKG2-interacting proteins common to both cell lines were identified (Fig. 3B and Supplementary Data 5–6). Notably, IGF2BP3 is a member of the IGF-2 mRNA-binding protein family that plays oncogenic roles in multiple cancers [27–30]. ranked among the top three most abundant proteins co-precipitated with PHKG2 (Fig. 3C, D). The findings were validated in cells by co-immunoprecipitation (co-IP) and proximity ligation assays (PLA) using antibodies against the PHKG2 and IGF2BP3 protein (Fig. 3E, F and Supplementary Fig. 3A, B). Immunofluorescence staining further revealed cytoplasmic co-localization of PHKG2 and IGF2BP3 in ESCC cell lines and patient-derived tumor tissues (Fig. 3G and Supplementary Fig. 3C).

Subsequently, an investigation was carried out to determine whether PHKG2 regulates IGF2BP3 expression. PHKG2 OE did not alter IGF2BP3 mRNA levels of IGF2BP3(Supplementary Fig. 3D) but significantly increased its protein abundance (Supplementary Fig. 3E). Conversely, knockout or knockdown of PHKG2 had no effect on IGF2BP3 transcription (Supplementary Fig. 3F, G) yet substantially reduced its protein levels (Supplementary Fig. 3H, I).

The phosphorylation dynamics of IGF2BP3 were then evaluated. PHKG2 OE increased the phosphorylation of IGF2BP3 in Eca109 cells (Fig. 3H), while PHKG2 KO or KD decreased phosphorylation in TE-1 cells (Fig. 3I), indicating that IGF2BP3 is a substrate of PHKG2. Mass spectrometry analysis identified threonine residues at positions 225 and 306 (T225/T306) as the phosphorylation sites of IGF2BP3 (Fig. 3J; Supplementary Fig. 3J and Supplementary Data 7). Site-directed mutagenesis was employed to assess phosphorylation-dependent regulation. Alanine substitutions at T225 (T225A) or T306 (T306A) partially reduced IGF2BP3 phosphorylation, whereas the double mutant (T225A/T306A, 2 A) completely abolished phosphorylation in 293 T cells (Supplementary Fig. 3K). In vitro kinase assay, PHKG2 interacted with IGF2BP3 and induced phosphorylation of serine/threonine (p - S/T) of IGF2BP3 (Fig. 3K).

Compared with wild-type (WT) IGF2BP3, the 2 A mutant exhibited enhanced ubiquitylation and accelerated protein degradation, while a phosphorylation-mimetic IGF2BP3 mutant (T225D/T306D, 2D) showed reduced ubiquitylation (Fig. 3L). The 2 A mutant also displayed significantly faster protein degradation than WT (Fig. 3M). Similarly, PHKG2 knockdown significantly accelerated IGF2BP3 protein degradation in KYSE-150 cells (Fig. 3N). These results suggested that PHKG2 regulates IGF2BP3 protein stability through ubiquitin-dependent proteasomal degradation. Furthermore, it was found that IGF2BP3 protein degradation was inhibited by treatment with the proteasome inhibitor MG132 in KYSE-150 and TE-1 cells (Fig. 3O and Supplementary Fig. 3L). Additionally, the reduction in IGF2BP3 protein levels induced by PHKG2 knockdown was rescued by MG132 treatment (Fig. 3P and Supplementary Fig. 3M). Consistent with this, increased IGF2BP3 ubiquitylation was observed in MG132-treated KYSE-150 cells with PHKG2 knockdown (Fig. 3Q). Collectively, these results demonstrate that PHKG2 stabilizes IGF2BP3 by phosphorylating T225 and T306, thereby inhibiting ubiquitin-dependent proteasomal degradation.

### PHKG2 promotes DDP resistance through IGF2BP3 in vitro and in vivo

**Fig. 4:**
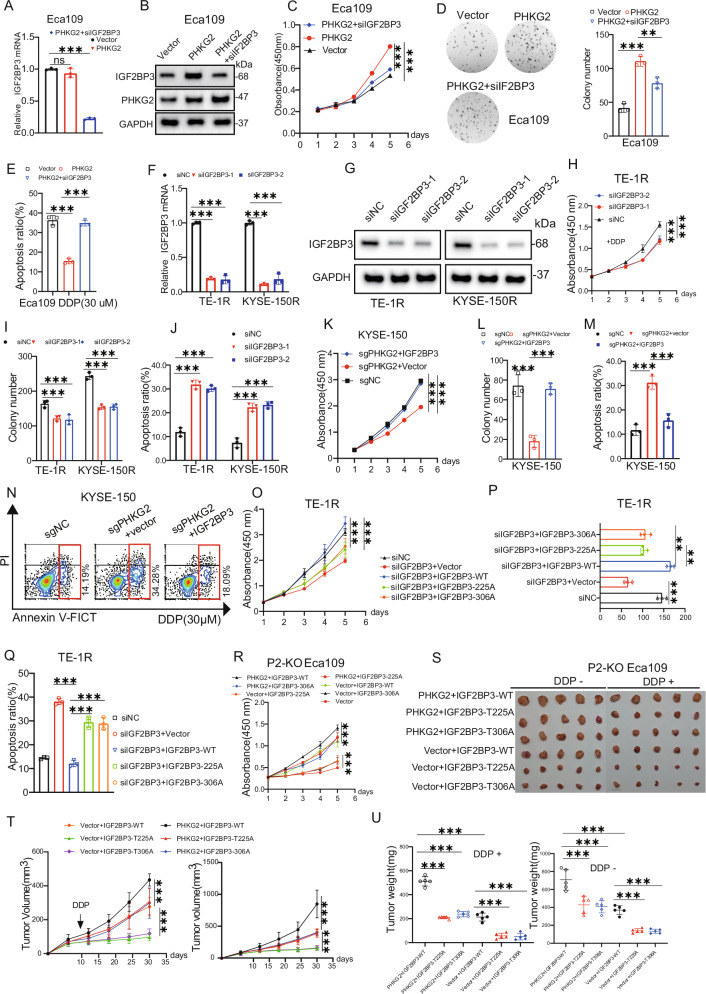
PHKG2 promotes DDP resistance through IGF2BP3 in vitro and in vivo. **A**, **B** IGF2BP3 expression levels in PHKG2-overexpressing Eca109 cells transfected with IGF2BP3 siRNA or control were determined by qPCR and western blot analysis(*n* = 3). **C**–**E** The impact of IGF2BP3 knockdown on cell proliferation (treated with 1 μM DDP) and apoptosis (treated with 30 μM DDP) was evaluated in PHKG2-overexpressing Eca109 cells(*n* = 3). **F**, **G** IGF2BP3 expression was analyzed by qPCR and western blot in TE-1-R and KYSE-150-R cells transfected with IGF2BP3 siRNA or control(*n* = 3). **H**, **I** The effect of IGF2BP3 knockdown on cell proliferation (1 μM DDP treatment) was assessed in TE-1-R and KYSE-150-R cells(*n* = 3). **J** Apoptosis (30 μM DDP treatment) was measured in TE-1-R and KYSE-150-R cells following IGF2BP3 knockdown(*n* = 3). **K**, **L** Cell proliferation (1 μM DDP) was evaluated in PHKG2-knockout KYSE-150 cells after IGF2BP3 overexpression(*n* = 3). **M**, **N** Apoptosis (30 μM DDP) was analyzed in PHKG2-knockout KYSE-150 cells with or without IGF2BP3 overexpression(*n* = 3). **O**, **P** The effect of overexpressing IGF2BP3 wild-type (WT), T225A, and T306A mutants on cell proliferation was assessed in IGF2BP3-knockdown TE-1-R cells and control cells treated with 1 μM DDP(*n* = 3). **Q** Apoptosis (30 μM DDP) was performed to compare IGF2BP3-WT and T225A/T306A mutant overexpression in IGF2BP3-knockdown TE-1-R cells(*n* = 3). **R** Proliferation of PHKG2-knockout Eca109 cells and PHKG2-knockout cells with PHKG2 overexpression was measured after introducing IGF2BP3-WT or T225A/T306A mutants, with cells treated with 1 μM DDP. **S** Subcutaneous xenograft tumors were established in nude mice (*n* = 5) using PHKG2-knockout Eca109 cells with or without PHKG2 overexpression and IGF2BP3-WT/T225A/T306A mutants, followed by treatment with 5 mg/kg DDP or vehicle. **T** Tumor volumes were measured to quantify the effect of IGF2BP3-WT and T225A/T306A mutants on tumor growth in PHKG2-knockout cells with or without PHKG2 overexpression under DDP treatment (*n* = 5). **U** The tumor weights were recorded at the experiment endpoint for PHKG2-knockout cells co-expressing PHKG2 and IGF2BP3-WT or T225A/T306A mutants (*n* = 5).

In light of our discovery that PHKG2 confers cisplatin resistance in ESCC, we postulated that the phosphorylation of IGF2BP3 might contribute to PHKG2-mediated DDP resistance. To verify this hypothesis, we knocked down IGF2BP3 in PHKG2-OE Eca109 cells (Fig. 4A, B) and analyzed their response to DDP treatment. The results indicated that IGF2BP3 KD significantly attenuated the proliferative advantage conferred by PHKG2 OE in Eca109 cells upon DDP exposure (Fig. 4C, D). Additionally, IGF2BP3 KD abrogated the anti-apoptotic effect of PHKG2 OE in DDP-treated Eca109 cells (Fig. 4E and Supplementary Fig. 4A). Furthermore, IGF2BP3 KD sensitized KYSE-150-R and TE-1-R cells to DDP-induced growth inhibition and apoptosis (Fig. 4F–J and Supplementary Fig. 4B–D). Conversely, IGF2BP3 OE significantly alleviated the suppressive effect of PHKG2 KO on KYSE-150 cell proliferation under DDP treatment (Fig. 4K, L and Supplementary Fig. 4E) and reversed the increased apoptosis caused by PHKG2 KO in DDP-exposed KYSE-150 cells (Fig. 4M, N).

To explore the functional role of phosphorylation at T225 and T306 in IGF2BP3, we compared the effects of overexpressing IGF2BP3 wild-type (WT), T225A mutant, and T306A mutant on cell proliferation and DDP-induced apoptosis in IGF2BP3-knockdown cells. Expression of IGF2BP3-WT fully rescued the proliferation defect caused by IGF2BP3 KD and reduced DDP-induced apoptosis in TE-1-R cells (Fig. 4O, P and Supplementary Fig. 4F, G). In contrast, T225A and T306A mutations significantly impaired the ability of IGF2BP3 to promote proliferation and suppress DDP-induced apoptosis (Fig. 4O, P and Supplementary Fig. 4F, G).

Given that T225 and T306 are threonine residues phosphorylated by PHKG2, we further investigated whether the function of IGF2BP3 is dependent on PHKG2-mediated phosphorylation. We assessed the effects of overexpressing IGF2BP3-T225A/T306A mutants versus IGF2BP3-WT on the response of PHKG2 KO Eca109 cells to DDP. The results demonstrated that T225A/T306A mutations significantly reduced the capacity of IGF2BP3 to promote proliferation and inhibit DDP-induced apoptosis in PHKG2 KO Eca109 cells (Fig. 4Q and Supplementary Fig. 4H). Similarly, co-expression of PHKG2 with IGF2BP3-T225A/T306A mutants had a less pronounced effect on proliferation and DDP-induced apoptosis compared with co-expression with IGF2BP3-WT (Fig. 4R and Supplementary Fig. 4H). To validate the in vivo significance of T225 and T306 phosphorylation in DDP resistance, we evaluated the impact of IGF2BP3-T225A/T306A mutations and PHKG2 co-expression on tumor growth in a subcutaneous xenograft model using PHKG2 KO Eca109 cells treated with DDP. Consistent with our in vitro findings, co-overexpression of PHKG2 and IGF2BP3-WT rescued the tumor growth defect in PHKG2-KO cells (Fig. 4S–U). Collectively, these findings suggest that phosphorylation of IGF2BP3 at T225 and T306 is essential for PHKG2-mediated DDP resistance in ESCC.

### PHKG2 and IGF2BP3 regulate CXCL8 expression in an m6A-dependent manner

**Fig. 5:**
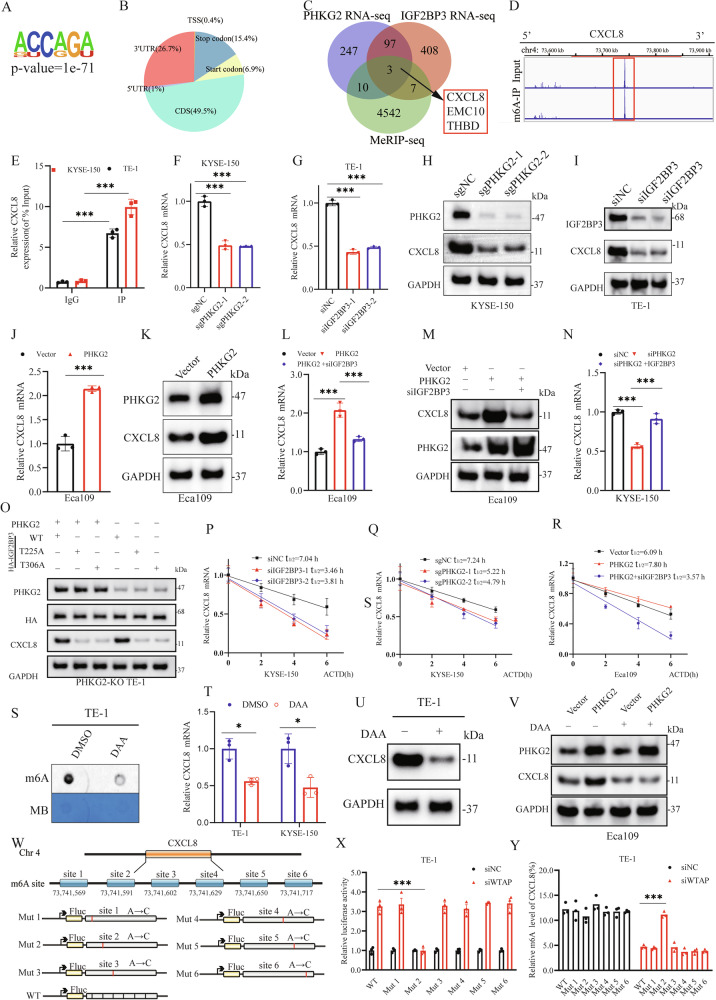
PHKG2 and IGF2BP3 regulate CXCL8 expression in an m6A-dependent manner. **A** Identification of the m6A consensus sequence motif in esophageal squamous cell carcinoma cells. **B** Distribution of N6-methyladenosine (m6A) modifications in messenger RNA (mRNA) transcripts. **C** Venn diagram integrating three datasets—genes upregulated in PHKG2-overexpressing cells, genes upregulated in IGF2BP3-overexpressing cells, and m6A-modified transcripts-to identify CXCL8, EMC10, and THBD as candidate IGF2BP3-regulated genes. **D** Integrative Genomics Viewer (IGV) plots depicting m6A modification peaks on CXCL8 mRNA from MeRIP-seq, showing subtracted read densities from m6A immunoprecipitation (IP) and input samples. **E** Direct binding of IGF2BP3 to CXCL8 transcript confirmed by RNA immunoprecipitation-quantitative PCR (RIP-qPCR) assay in TE-1 and KYSE-150 cells(*n* = 3). **F**, **G** qPCR analysis of CXCL8 mRNA expression in PHKG2-knockout, IGF2BP3-knockdown, and control KYSE-150 and TE-1 cells(*n* = 3). **H**, **I** Western blot analysis of CXCL8 protein levels in PHKG2-KO, IGF2BP3-KD, and control KYSE-150 and TE-1 cells. **J** qPCR analysis of CXCL8 mRNA expression in PHKG2-overexpressing Eca109 cells compared to control cells(*n* = 3). **K** WB analysis of CXCL8 protein expression in PHKG2-OE and control Eca109 cells. **L**, **M** CXCL8 mRNA and protein levels in control, PHKG2-OE, and PHKG2-OE + IGF2BP3-KD Eca109 cells, demonstrating rescue by IGF2BP3 knockdown(*n* = 3). **N** CXCL8 protein expression in control, PHKG2-KD, and PHKG2-KD + IGF2BP3-OE KYSE-150 cells. **O** WB analysis of CXCL8 protein levels in PHKG2-KO TE-1 cells infected with lentiviruses expressing PHKG2 + IGF2BP3-wild-type (WT), PHKG2 + IGF2BP3-T225A, PHKG2 + IGF2BP3-T306A, vector + IGF2BP3-WT, or vector + IGF2BP3-T225A/T306A mutants. **P**, **Q** Assessment of CXCL8 mRNA stability via actinomycin D chase assay in IGF2BP3-KD and PHKG2-KO KYSE-150 and TE-1 cells(*n* = 3). **R** CXCL8 mRNA half-life analysis in PHKG2-OE Eca109 cells transfected with IGF2BP3 siRNA or control, measured by actinomycin D treatment(*n* = 3). **S** Dot blot analysis of global m6A levels in TE-1 cells treated with 50 μM 3-deazaadenosine (DAA) or DMSO control, with methylene blue (MB) staining as a loading control. **T** qPCR analysis of CXCL8 mRNA expression in KYSE-150 and TE-1 cells treated with 50 μM DAA(*n* = 3). **U** WB analysis of CXCL8 protein levels in TE-1 cells treated with 50 μM DAA or vehicle control. **V** CXCL8 protein expression in PHKG2-OE and control cells treated with 50 μM DAA or vehicle, assessing m6A-dependent regulation. **W** Schematic illustration of the localization of m6A motifs and their respective mutant sites. **X** Validation of six m6A modification sites via stepwise mutation-based luciferase reporter assays(*n* = 3). **Y** Confirmation of six m6A modification sites using Me-RIP-qPCR(*n* = 3).

Considering that IGF2BP3 serves as a reader of N⁶-methyladenosine (m6A), the most abundant RNA modification in eukaryotic cells, and promotes the stability of m6A-modified transcripts [31]. We aimed to identify downstream effectors mediating DDP resistance in ESCC using m6A-specific methylated RNA immunoprecipitation sequencing(MeRIP-seq). MeRIP-seq analysis in KYSE-150-R cells identified 4,564 genes with significantly increased m6A modification (m6A single site ≥5; fold enrichment ≥4) compared with input controls (Supplementary Fig. 5A–C and Supplementary Data 8). We identified a consensus m6A sequence motif (Fig. 5A) and discovered that m6A modifications were predominantly localized in coding sequences (CDS, 49.5%), 3′ untranslated regions (3′ UTR, 26.7%), and stop codons (15.4%) of chromosome 1-encoded genes (Fig. 5B and Supplementary Fig. 5A, B). Gene ontology (GO) enrichment analysis indicated that m6A-modified genes were significantly associated with mRNA processing, RNA splicing, and mRNA splicing pathways (Supplementary Fig. 5C).

To identify genes regulated by IGF2BP3, we performed RNA sequencing (RNA-seq) in IGF2BP3-overexpressing(OE) TE-1 cells and control cells, concentrating on significantly upregulated transcripts (Supplementary Fig. 5D and Supplementary Data 9). Concurrently, RNA-seq of PHKG2-OE TE-1 cells identified PHKG2-regulated genes (Supplementary Fig. 5E and Supplementary Data 10). Integrative analysis of m6A-modified transcripts, IGF2BP3-upregulated genes, and PHKG2-upregulated genes highlighted chemokine ligand 8(CXCL8), endoplasmic reticulum membrane protein complex subunit 10(EMC10), and thrombomodulin (THBD) as potential IGF2BP3 targets (Fig. 5C). Among these, CXCL8—unlike EMC10 or THBD—has been directly linked to cancer cell survival, metastasis, and DDP resistance in multiple tumor types [32–35]. leading us to prioritize it for functional validation.

It was confirmed that CXCL8 mRNA to contains m6A modifications (Fig. 5D) and interacts with IGF2BP3 in TE-1 and KYSE-150 cells (Fig. 5E). PHKG2 and IGF2BP3 positively regulated CXCL8 expression: PHKG2 knockout (KO) or IGF2BP3 knockdown (KD) reduced the levels of CXCL8 mRNA and protein in KYSE-150, TE-1, and TE-1-R cells (Fig. 5F–I and Supplementary Fig. 5F–I), whereas PHKG2-OE or IGF2BP3-OE enhanced CXCL8 expression in Eca109 cells (Fig. 5J, K and Supplementary Fig. 5J, K). Crucially, IGF2BP3-KD abrogated the upregulation of CXCL8 in PHKG2-OE Eca109 cells (Fig. 5L, M), while IGF2BP3-OE rescued CXCL8 downregulation in PHKG2-KD KYSE-150 cells (Fig. 5N and Supplementary Fig. 5L), indicating that PHKG2 regulates CXCL8 via IGF2BP3.

To confirm the role of PHKG2-mediated phosphorylation, we overexpressed PHKG2 in PHKG2-KO TE-1 cells expressing HA-tagged IGF2BP3 wild-type(WT), T225A, or T306A mutants. CXCL8 expression was significantly increased by PHKG2-OE in IGF2BP3-WT cells but not in cells expressing phosphorylation-deficient mutants (Fig. 5O), validating that the phosphorylation of IGF2BP3 at T225/T306 is essential for CXCL8 regulation. Given IGF2BP3’s role in stabilizing m6A-modified mRNAs [31]. We examined the stability of CXCL8 mRNA in IGF2BP3 KD cells. As expected, IGF2BP3 KD significantly reduced the half-life of CXCL8 mRNA in KYSE-150 and TE-1 cells (Fig. 5P and Supplementary Fig. 5M). PHKG2 KO also reduced the stability of CXCL8 mRNA (Fig. 5Q and Supplementary Fig. 5N), while PHKG2 OE prolonged the half-life of CXCL8 mRNA, an effect reversed by IGF2BP3 KD in PHKG2-OE cells (Fig. 5R). Additionally, treatment with 3-deazaadenosine (DAA), an m6A inhibitor, significantly decreased global m6A levels in KYSE-150 and TE-1 cells (Fig. 5S and Supplementary Fig. 5O) and reduced CXCL8 mRNA and protein expression (Fig. 5T, U and Supplementary Fig. 5P), suggesting m6A is critical for the stability of CXCL8 mRNA. DAA treatment also downregulated the expression of CXCL8 induced by PHKG2 OE (Fig. 5V).

To understand the effects of m6A modification on CXCL8 expression, the expression of m6A writers was then evaluated in DDP-P ESCC and DDP-R ESCC cells. Among these m6A writers, WTAP showed remarkably increased expression in DDP-R cells, which could account for the upregulated m6A levels (Supplementary Fig. 5Q). WTAP knockdown in ESCC cells was then constructed (Supplementary Fig. 5R). CXCL8 was significantly downregulated by WTAP knockdown (Supplementary Fig. 5S). WTAP knockdown significantly decreased the stability of CXCL8 mRNA (Fig. 5T). To further search for m6A sites in CXCL8 mRNA, we generated dual-luciferase reporter constructs containing firefly luciferase before the wild-type or mutant CXCL8 mRNA sequence with A at the m6A sites substituted with C (Fig. 5W). The results of luciferase reporter assays showed that the site2 mutation decreased the m6A modification of CXCL8 mRNA after WTAP knockdown (Fig. 5X). These results were confirmed by Me-RIP-qPCR (Fig. 5Y). Collectively, these results revealed that the stability of CXCL8 mRNA was regulated by PHKG2-mediated phosphorylation of IGF2BP3 in an m6A-dependent manner.

### PHKG2 and IGF2BP3 regulate CXCL8 expression in IGF2BP3 phase separation manner

Numerous RNA-binding proteins have been reported to engage in liquid‒liquid phase separation, giving rise to liquid‒like droplets that govern the fate of target RNAs [36–41]. However, whether IGF2BP3 undergoes phase separation remains unclear. To further explore how IGF2BP3 regulates m⁶A-modified RNAs, we investigated the extent of IGF2BP3 condensation in ESCC cells.

**Fig. 6:**
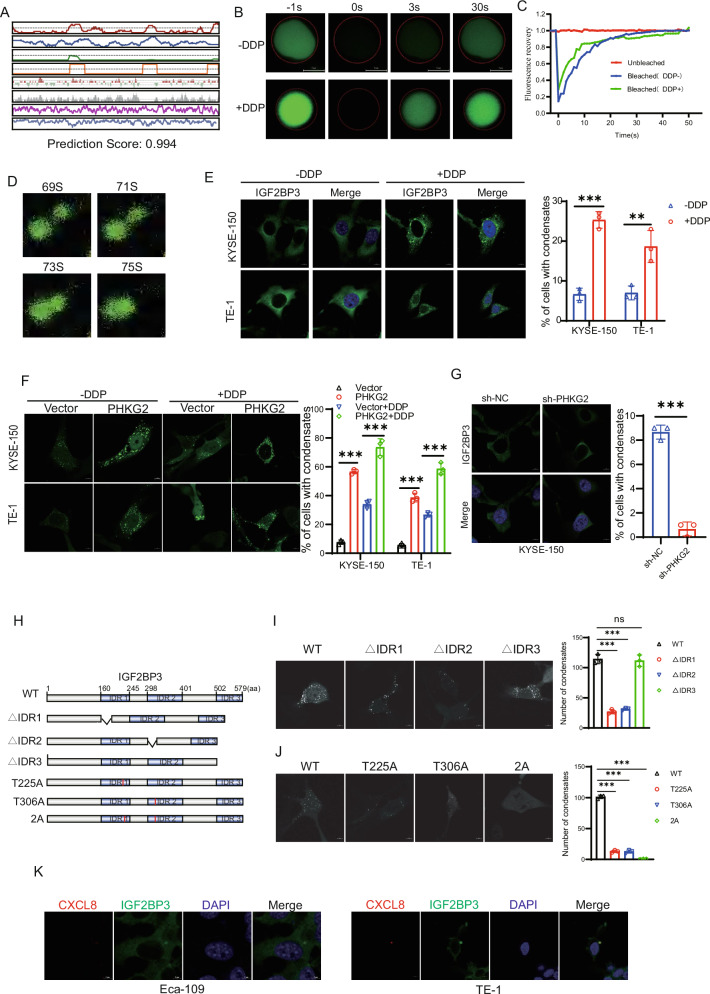
PHKG2 and IGF2BP3 regulate CXCL8 expression in an IGF2BP3 phase separation manner. **A** The comprehensive prediction score for IGF2BP3 undergoing phase separation was 0.994 as determined by prediction tools. **B** Representative images of fluorescence recovery and quantitative analysis of FRAP assay results are shown (Scale bar, 2 μm). **C** Quantitative analysis of the FRAP assay. **D** IGF2BP3-GFP-labeled droplets merged to form larger droplets in TE-1R cell. **E** IF staining confirmed the condensation of IGF2BP3 in ESCC cells(*n* = 3). **F** PHKG2 overexpression induced phase separation of the IGF2BP3 protein in ESCC cells(*n* = 3). **G** PHKG2 knockdown suppressed the phase separation of the IGF2BP3 protein in ESCC cells(*n* = 3). **H** Schematic diagram of the IGF2BP3 mutants. **I** Representative images of ESCC cells overexpressing IGF2BP3 deletion mutants(*n* = 3). **J** Representative images of ESCC cells overexpressing IGF2BP3 mutants(*n* = 3). **K** RNA fluorescence in situ hybridization (FISH) combined with immunofluorescence (IF) was used to detect the colocalization of CXCL8 mRNA and IGF2BP3 condensates.

Initially, through the utilization of prediction tools [42]. It was discovered that the comprehensive score for IGF2BP3 to undergo phase separation was 0.994, strongly suggesting a high probability of phase separation (Fig. 6A). Subsequently, fluorescence recovery after photobleaching (FRAP) assays were conducted. The results of these assays indicated that the fluorescence signal of IGF2BP3-GFP recovered after bleaching, regardless of the presence or absence of DDP (Fig. 6B, C). Additionally, it was observed that two IGF2BP3-GFP droplets could coalesce into a larger droplet (Fig. 6D). Immunofluorescence (IF) staining verified the condensation of IGF2BP3 in KYSE-150 and TE-1 cell lines. Notably, in the presence of DDP, the puncta in KYSE-150 and TE-1 were significantly more prominent compared with the situation without DDP. (Fig. 6E). To ascertain the impact of phosphorylation on the phase separation of IGF2BP3, we assessed the alterations in the number of IGF2BP3 puncta subsequent to PHKG2 overexpression (OE) in TE - 1 and KYSE - 150 cells. PHKG2 OE notably promoted the formation of IGF2BP3 puncta, regardless of the presence or absence of cisplatin (Fig. 6F). Conversely, PHKG2 KD significantly disrupted IGF2BP3 puncta formation (Fig. 6G).

Intrinsically disordered regions (IDRs) are recognized as being critical for phase separation [43]. By employing phase separation predictors [44]. Three putative IDRs were identified in IGF2BP3 (Fig. 6H), which may facilitate the formation of condensates. To characterize these domains, we generated IGF2BP3 deletion mutants: ∆IDR1, ∆IDR2 and ∆IDR3 (Fig. 6H). Reconstitution assays with purified mutants from transfected IGF2BP3-KD ESCC cells in the presence of 10% PEG showed that ∆IDR1 and ∆IDR2 (but not ∆IDR3) attenuated condensate formation, demonstrating that IDR1 and IDR2 are crucial for IGF2BP3 phase separation(Fig. 6I).

Further investigation was conducted to determine whether phosphorylation site mutations in IDR1 and IDR2 affect IGF2BP3 phase separation. T225A and T306A mutations significantly reduced phase separation, while the T225A/T306A (2 A) double mutant completely abolished it (Fig. 6J). These findings indicate that PHKG2-mediated phosphorylation of IGF2BP3 is critical for its phase separation. Furthermore, RNA fluorescence in situ hybridization assays showed that CXCL8 mRNA colocalized with cytoplasmic IGF2BP3 condensates (Fig. 6K). Collectively, these results revealed that PHKG2 regulates CXCL8 expression in an IGF2BP3 phase separation-dependent manner.

### The effect of PHKG2 and IGF2BP3 on DDP resistance is dependent on CXCL8

**Fig. 7:**
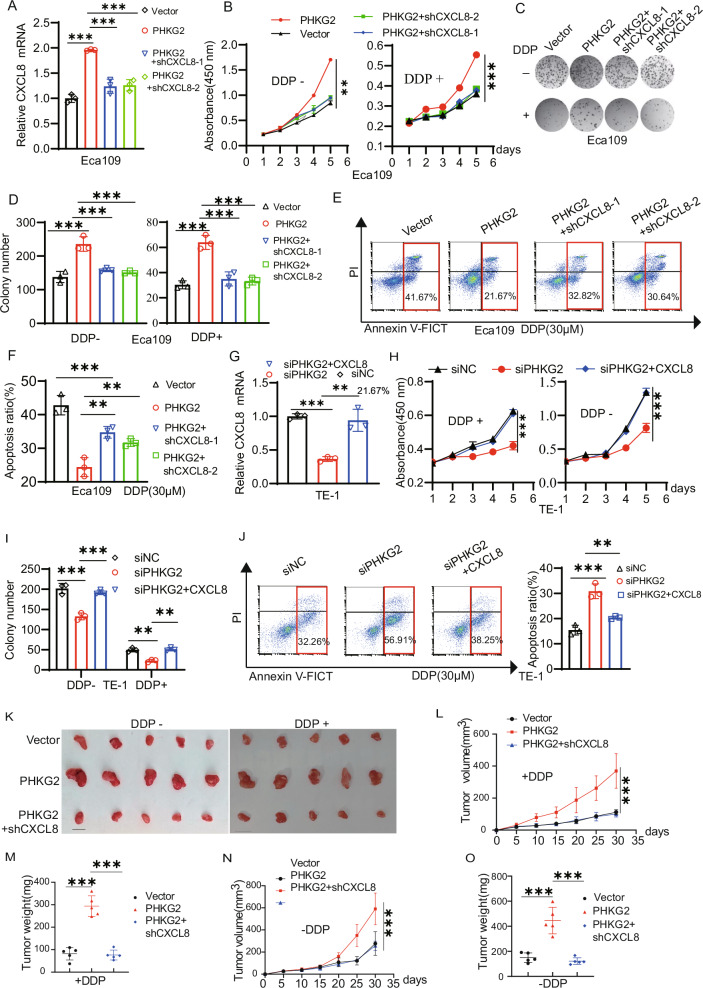
The effect of PHKG2 and IGF2BP3 on DDP resistance is dependent on CXCL8. **A** qPCR confirmed efficient knockdown of CXCL8 using two independent shRNAs(*n* = 3). **B** CCK-8 assay showed the effect of CXCL8 knockdown on the proliferation of PHKG2-overexpressing Eca109 cells treated with 1 μM DDP. **C**, **D** Colony formation assays evaluated the impact of CXCL8 knockdown on the proliferative capacity of PHKG2-overexpressing Eca109 cells under 1 μM DDP treatment(*n* = 3). **E**, **F** Flow cytometry analysis assessed the effect of CXCL8 knockdown on apoptosis in PHKG2-overexpressing Eca109 cells treated with 30 μM DDP(*n* = 3). **G** qPCR confirmed successful overexpression of CXCL8 in PHKG2-knockdown TE-1 cells(*n* = 3). **H** CCK-8 assay determined the effect of CXCL8 overexpression on the proliferation of PHKG2-knockdown TE-1 cells treated with 1 μM DDP. **I** Colony formation assays analyzed the impact of CXCL8 overexpression on the proliferative ability of PHKG2-knockdown TE-1 cells under 1 μM DDP treatment(*n* = 3). **J** Flow cytometry analysis examined the effect of CXCL8 overexpression on apoptosis in PHKG2-knockdown TE-1 cells treated with 30 μM DDP(*n* = 3). **K**, **L**, **N** The effect of CXCL8 KD on the tumor growth of Eca109 cells overexpressing PHKG2 treated with DDP (5 mg/kg) or vehicle, as shown by measurement of tumor volume (*n* = 6). **M**, **O** The effect of CXCL8 KD on the tumor growth of Eca109 cells overexpressing PHKG2 treated with DDP (5 mg/kg) or vehicle, as shown by tumor weights at the endpoint (*n* = 6).

Considering that CXCL8 is regulated by PHKG2, we explored whether CXCL8 serves as a downstream effector of PHKG2 in mediating DDP resistance. CXCL8 KD in Eca109 cells (Fig. 7A and Supplementary Fig. 6A) abrogated the enhanced cell proliferation and colony formation capacity induced by PHKG2 OE under DDP treatment (Fig. 7B–D). Similarly, CXCL8 KD reversed the pro-proliferative and colony-forming effects of PHKG2 OE even in the absence of DDP (Fig. 7B–D). Moreover, CXCL8 KD restored sensitivity to DDP-induced apoptosis in PHKG2-OE Eca109 cells (Fig. 7E, F). In PHKG2-KD TE-1 cells, CXCL8 OE (Fig. 7G and Supplementary Fig. 6B) rescued deficiencies in cell proliferation and colony formation under both DDP exposure and normal culture conditions(Fig. 7H, I and Supplementary Fig. 6C). CXCL8 OE also counteracted the increased apoptosis caused by PHKG2 knockout in DDP-treated TE-1 cells (Fig. 7J). In DDP-resistant TE-1-R cells, CXCL8 KD (Supplementary Fig. 6D, E) resulted in decreased cell proliferation (Supplementary Fig. 6F, G), reduced colony formation (Supplementary Fig. 6H), and enhanced DDP-induced apoptosis (Supplementary Fig. 6I). In vivo, CXCL8 KD markedly abrogated DDP resistance induced by PHKG2 OE in Eca109 cells (Fig. 7K), as evidenced by reduced tumor volumes (Fig. 7L) and weights (Fig. 7M) at the endpoint. CXCL8 KD also suppressed PHKG2-induced ESCC growth in the absence of DDP treatment in vivo (Fig. 7N, O). To assess the impact of CXCL8 KD on cancer cell proliferation in vivo, we performed Ki67 IHC staining on tumor sections (Supplementary Fig. 6J). CXCL8 KD in PHKG2-OE Eca109 cells significantly reduced the proportion of Ki67⁺ cells and markedly increased the ratio of cleaved caspase-3⁺ cells compared with PHKG2-OE Eca109 cells, both with and without DDP treatment (Supplementary Fig. 6K, L). Collectively, these results indicated that CXCL8 plays a critical role in the PHKG2-mediated DDP resistance in ESCC cells.

### High expression of PHKG2 in ESCC cells is closely related to M2 polarization of macrophages

**Fig. 8:**
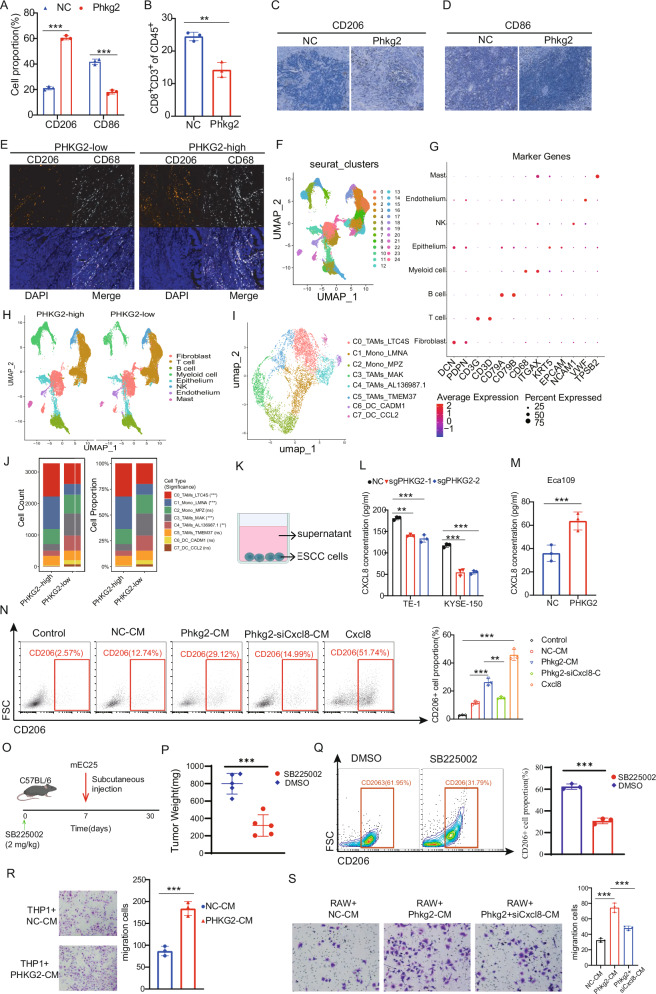
High expression of PHKG2 in ESCC cells is closely related to M2 polarization of macrophages. **A** Quantitative analysis of CD206+ or CD86+ cells in subcutaneous tumors of Phkg2-OE group and control group by flow cytometry(*n* = 3). **B** Quantitative analysis of CD3 + CD8+ of CD 45+ cells in subcutaneous tumors of PHKG2-OE group and control group by flow cytometry(*n* = 3). **C**, **D** IHC analysis of CD206+ and CD86+ cells in subcutaneous tumors of Phkg2-OE group and control group. **E** Multiplex IF staining showing the expression levels of CD206 and CD68 in human ESCC. **F** UMAP plot of all cell clusters from single-cell RNA sequencing. **G** Expression levels of various marker genes in different cell subpopulations. **H** UMAP plots of all cell clusters in PHKG2 low-expression group and PHKG2 high-expression group. **I**, **J** Single-cell RNA sequencing UMAP plots of all tumor-associated macrophage clusters. **K** Schematic diagram of ESCC Supernatant. **L** ELISA assay showing the expression level of CXCL8 in cell culture supernatants of PHKG2-KD group and control group in TE-1 and KYSE-150 cells(*n* = 3). **M** ELISA assay showing the expression levels of CXCL8 in cell culture supernatants of PHKG2-OE group and control group in Eca109 cells (*n* = 3). **N** Flow cytometry showing the effect of PHKG2 and CXCL8 on the differentiation ability of mouse bone marrow-derived macrophages (BMDMs, *n* = 3). **O** Schematic of the ESCC xenograft model treated with SB225002(2 mg/kg). **P** The effect of CXCL8 inhibition on the tumor growth of mEC25 cells treated with SB225002 or vehicle, as shown by tumor weights at the endpoint (*n* = 5). **Q** Quantitative analysis of CD206+ cells in subcutaneous tumors by flow cytometry. **R** Transwell assay showing the invasive ability of THP-1 cells co-cultured with supernatants from PHKG2-OE and control TE-1 cells group (*n* = 3). **S** Transwell assay showing the invasive ability of RAW cells co-cultured with supernatants from different mEC25 cells (*n* = 3).

Accumulating evidence indicates that CXCL8, as a downstream effector of PHKG2, recruits tumor-associated macrophages (TAMs) to alter the tumor microenvironment and promote cancer progression [45]. Consequently, we explored whether PHKG2 contributes to such alterations in the immune microenvironment. Subcutaneous tumorigenesis assays were performed using control and Phkg2-overexpressing mEC25 cells, followed by flow cytometric analysis of intratumoral macrophages and T cells. Tumors with elevated Phkg2 levels showed a significant reduction in M1-phenotype macrophages (CD86⁺) and a concurrent increase in M2-phenotype macrophages (CD206⁺) (Fig. 8A). Additionally, Phkg2 expression negatively correlated with intratumoral CD8⁺ T cell levels (Fig. 8B). IHC staining of subcutaneous tumors further validated these findings, confirming corresponding changes in the M1/M2 macrophage ratio (Fig. 8C, D). Multiplex immunofluorescence (IF) staining of human ESCC tissues revealed a positive correlation between PHKG2 expression and M2 macrophage abundance (Fig. 8E). Analysis of single-cell sequencing data from a public database (GSE209524) further confirmed a positive correlation between PHKG2 expression and the proportion of M2 macrophages (Fig. 8F–J). Consistent with our earlier findings that PHKG2 significantly upregulates CXCL8 mRNA levels in ESCC cells, PHKG2 overexpression or knockdown markedly increased or decreased CXCL8 concentrations in cell culture supernatants, respectively (Fig. 8K–M). Co-culturing supernatants from ESCC cells with mouse bone marrow-derived macrophages (BMDMs) showed that conditioned medium from PHKG2-overexpressing cells induced BMDM differentiation toward an M2 phenotype, whereas CXCL8 knockdown in PHKG2-overexpressing cells abrogated this effect (Fig. 8N). To further validate the role of CXCL8 in the proportion of M2 macrophages, mEC25-bearing mice were treated with the CXCL8 receptor antagonist SB225002(Fig. 8O). The findings indicated that the inhibition of CXCL8 notably decreased tumor weights and the quantity of M2-phenotype macrophages(Fig. 8P, Q). Transwell assays further demonstrated that PHKG2 enhances macrophage migration via CXCL8 (Fig. 8R, S). Collectively, these results indicate that PHKG2 promotes M2 macrophage polarization through CXCL8, leading to functional suppression of CD8⁺ T cells and ultimately contributing to the establishment of an immunosuppressive tumor microenvironment.

### Clinical significance of targeting PHKG2 in ESCC

**Fig. 9:**
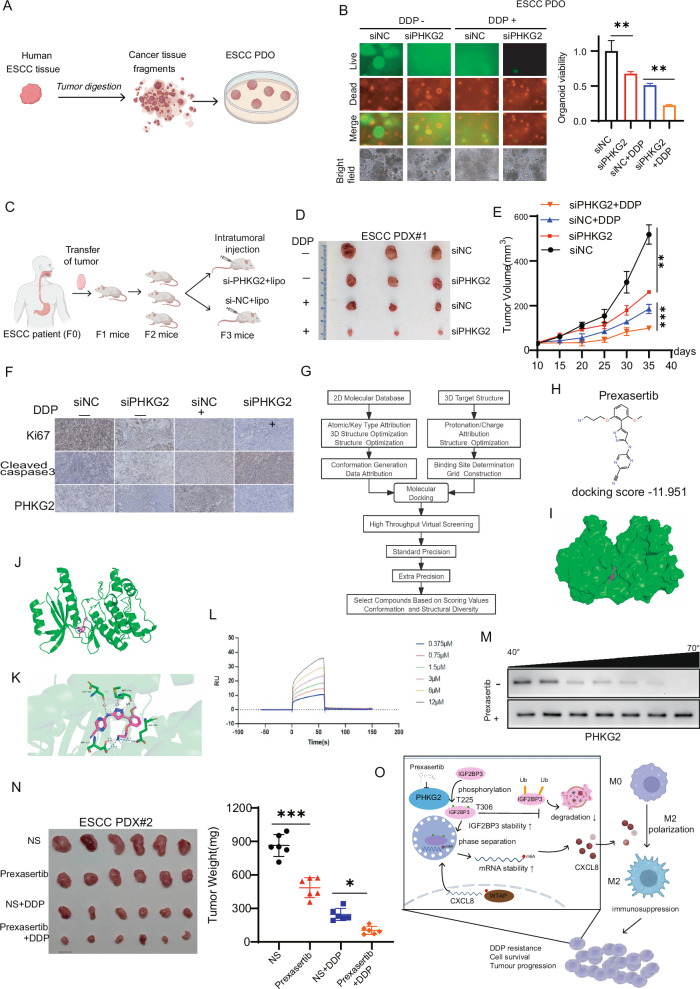
Clinical significance of targeting PHKG2 in ESCC. **A** Schematic diagram illustrating the workflow of generating ESCC patient-derived organoid(PDO) models. **B** Representative fluorescence and bright-field microscopy images of ESCC patient-derived organoids, with cell viability assessed by CellTiter-Glo assay (*n* = 3). **C** Schematic diagram depicting the workflow of establishing ESCC patient-derived xenograft(PDX) models. **D** Tumor images of extracted ESCC PDX transduced with siNC or siPHKG2, with or without DDP treatment(*n* = 3). **E** Tumor growth curves of ESCC PDX models across experimental groups(*n* = 3). **F** Quantitative analysis of Ki67 and cleaved caspase 3 expression in ESCC PDX tumor tissues (*n* = 3). **G** Flowchart of virtual screening. **H** The chemical structure of prexasertib. **I**–**K** Cartoon, surface and 3D diagrams of prexasertib and PHKG2 protein molecules. **L** The human PHKG2 proteins were prepared and binding with prexasertib was assayed with SPR. **M** Binding of prexasertib with PHKG2 protein was detected with the CETSA analysis. Cells were treated with vehicle or prexasertib at 40, 45, 50, 55, 60, 65 and 70 °C, and PHKG2 expression was determined by western blotting. **N** Tumor image and tumor weight of extracted ESCC PDX treated with or without prexasertib at the endpoint of the experiment(*n* = 6). **O** Schematic illustration of the underlying mechanisms of PHKG2 in promoting DDP resistance in ESCC.

To further validate PHKG2 as a therapeutic target, we developed ESCC patient-derived organoid (PDO) models and knocked down PHKG2 using siRNA(Fig. 9A). PHKG2 knockdown significantly inhibited PDO formation and enhanced DDP sensitivity in these models(Fig. 9B). To further confirm the significance of targeting PHKG2 in vivo, we established ESCC patient-derived xenograft (PDX) models in nude mice(Fig. 9C). The combination of siRNA-mediated PHKG2 knockdown and DDP treatment markedly suppressed tumor growth (Fig. 9D, E and Supplementary Fig. 7A). IHC analysis showed that PHKG2 knockdown increased cleaved caspase-3 levels and reduced the proportion of Ki67⁺ cells, with more pronounced effects under DDP exposure (Fig. 9F and Supplementary Fig. 7B, C). Based on these PDO and PDX findings, it was hypothesized that targeting PHKG2 could improve ESCC treatment and overcome cisplatin resistance.

To preclinically validate this, we identified potential PHKG2 inhibitors via virtual screening of 306,100 small-molecule compounds against PHKG2’s catalytic pocket (Fig. 9G). The top 200 hits were selected and tested for in vitro PHKG2 inhibitory activity (Supplementary Data 8). Notably, prexasertib, a CHK1 inhibitor used in multiple clinical trials [46–48]. emerged as a candidate with high binding affinity to PHKG2’s catalytic pocket (Fig. 9H). 2D and 3D binding analyses showed that the two imino groups of prexasertib formed hydrogen bonds with MET110 and ASP108 of PHKG2, respectively (Fig. 9I–K and Supplementary Fig. 7D). The direct association between prexasertib and purified PHKG2 protein was confirmed with the SPR assay (Fig. 9L). In addition, the thermal shift assay showed that prexasertib could also bind to PHKG2 in ESCC cells (Fig. 9M). In an ESCC PDX model, prexasertib treatment significantly inhibited tumor growth (Fig. 9N and Supplementary Fig. 7E). IHC analysis confirmed increased cleaved caspase-3 levels and reduced Ki67⁺ cells in prexasertib-treated tumors(Supplementary Fig. 7F–H). Collectively, these results indicate that PHKG2 represents a promising therapeutic target for overcoming cisplatin resistance in ESCC.

## Discussion

ESCC persists as a formidable global health challenge, with high mortality rates despite advances in treatment modalities [3]. DDP-based chemotherapy regimens have been extensively adopted as neoadjuvant, adjuvant, and palliative therapies for ESCC over several decades [49, 50]. However, patients with ESCC often have a poor prognosis due to therapeutic resistance, and the molecular mechanisms underlying DDP resistance in ESCC cells remain largely elusive.

Our study identified the PHKG2-IGF2BP3-CXCL8-M2 macrophage axis as a key regulator of DDP resistance in ESCC, suggesting that targeting PHKG2 may be an effective strategy to enhance ESCC responsiveness to DDP. Protein phosphorylase kinases have emerged as attractive therapeutic targets in cancer [15]. and a deeper understanding of how these enzymes drive tumor progression and therapeutic resistance provides a rationale for their targeting in oncology.

Previous studies have implicated PHKG2 in ferroptosis, tumor growth, and metabolism, but its role in mediating DDP resistance remained unclear. Utilizing genome-wide CRISPR screening combined with RNA sequencing of DDP-resistant cell lines and clinical specimens from patients with differential DDP responses, we identified PHKG2 as a potential driver of DDP resistance, with functional validation in vitro and in vivo. Our screening also identified additional candidate genes associated with DDP resistance (Fig. 1D and Supplementary Tables 2, 3, and 4). Among these, genes such as ENO3, KIF22, CDCA8, TAP1, and ORC6 have known oncogenic roles in tumorigenesis [51–55]. while SPP1 has been linked to therapeutic resistance [56]. Given that therapeutic resistance can arise through multiple independent pathways, functional validation of these candidates and assessment of their clinical relevance are warranted, as they may offer additional targets to overcome DDP resistance.

Phosphorylation is a key regulator of protein function [15]. Our study identified IGF2BP3 as a critical substrate of PHKG2: PHKG2 stabilizes IGF2BP3 by phosphorylating it at T225 and T306, and this interaction mediates PHKG2-driven DDP resistance in vitro and in vivo. Our proteomic analysis also revealed multiple PHKG2-interacting proteins that may serve as its substrates (Fig. 3D and Supplementary Tables 5 and 6). Notably, ELAVL1—one of the top interactors—has been implicated in regulating ferroptosis and autophagy, processes linked to therapeutic resistance [57]. Future studies should investigate whether ELAVL1 is a bona fide PHKG2 substrate and whether its phosphorylation contributes to DDP resistance. To evaluate the metabolic function of PHKG2, a metabolomic analysis was performed on TE - 1 cells. The analyses were conducted on stable cell lines of two genotypes: the wild-type (WT) with an empty vector and the PHKG2 knockdown, under cisplatin stress. It was identified that S - Adenosylhomocysteine exhibited a significant decrease in the PHKG2 knockdown group compared with the WT cells (Supplementary Fig. 7I). Previous research has demonstrated that S - adenosylhomocysteine (SAH) can significantly reduce the total m6A levels [58–60], which implies that PHKG2 may regulate m⁶A levels through modulating SAH metabolism.

IGF2BP3, a member of the IGF2BP family (including IGF2BP1, IGF2BP2, and IGF2BP3), regulates chemotherapy resistance by stabilizing oncoprotein mRNAs [24]. Recent work showed that IGF2BP3 promotes SENP1 expression via m⁶A modification [23]. Through integrated MeRIP-seq and transcriptomic analyses, we identified CXCL8, EMC10, and THBD as m⁶A-modified transcripts targeted by IGF2BP3. Our data highlight the critical role of T225/T306 phosphorylation in IGF2BP3 function, and RNA FISH confirmed CXCL8 mRNA colocalization with cytoplasmic IGF2BP3 condensates. While we focused on CXCL8 as a key effector, EMC10 and THBD may also contribute to DDP resistance, warranting future investigation into their roles as IGF2BP3 targets.

Numerous studies have shown that CXCL8 recruits tumor-associated macrophages (TAMs), perturbing the tumor microenvironment and offering therapeutic opportunities [45, 61]. However, the association between PHKG2 and the immune microenvironment was unreported. Our study is the first to link PHKG2 to TAM regulation: PHKG2 enhances CXCL8 expression and secretion in ESCC cells, inducing M0-to-M2 macrophage polarization in the tumor immune microenvironment. M2 macrophages suppress CD8⁺ T cell cytotoxicity, fostering an immunosuppressive microenvironment.

Clinically, high PHKG2 expression strongly correlates with poor prognosis and reduced response to DDP-based neoadjuvant therapy in ESCC patients. Using ESCC PDX models, we validated PHKG2’s therapeutic potential: by analyzing PHKG2’s protein crystal structure and screening the MCE small-molecule library, we identified prexasertib as a candidate inhibitor, which significantly suppressed PDX tumor growth. These findings support PHKG2 as a promising therapeutic target for overcoming cisplatin resistance.

Our study has limitations. First, our CRISPR screening lacked biological replicates, reducing robustness compared to replicated screens; some enriched gRNAs may have emerged by chance. Additional hits will be validated through functional studies or a secondary CRISPR screen. Second, our single-center cohort limits the generalizability of PHKG2 as a DDP response biomarker, necessitating validation in larger multi-center cohorts with more PDX and PDO models. Third, since DDP-based neoadjuvant therapy is often combined with paclitaxel, future studies should explore whether PHKG2 also modulates ESCC responsiveness to paclitaxel.

## Conclusion

This study highlights the promising potential of targeting PHKG2 as a therapeutic strategy for overcoming cisplatin resistance in ESCC.

## Supplementary information

Supplementary Figures and Tables.

uncropped blots Fig

Supplementary Data 1

Supplementary Data 2

Supplementary Data 3

Supplementary Data 4

Supplementary Data 5

Supplementary Data 6

Supplementary Data 7

Supplementary Data 8

Supplementary Data 9

Supplementary Data 10

Supplementary Data 11

## Data Availability

The raw sequence data reported in this paper have been deposited in the Genome Sequence Archive (Genomics, Proteomics & Bioinformatics 2025) in the National Genomics Data Center (Nucleic Acids Res 2025), China National Center for Bioinformation / Beijing Institute of Genomics, Chinese Academy of Sciences (GSA-Human: HRA015661), which are publicly accessible at https://ngdc.cncb.ac.cn/gsa-human. All data pertinent to this study, whether generated or analyzed, are comprehensively presented in this manuscript and its supplementary information. For any additional inquiries or requests, interested parties are encouraged to contact the corresponding authors.

## References

[1] Bray F, Laversanne M, Sung H, Ferlay J, Siegel RL, Soerjomataram I, et al. Global cancer statistics 2022: GLOBOCAN estimates of incidence and mortality worldwide for 36 cancers in 185 countries. CA Cancer J Clin. 2024;74:229–63. 10.3322/caac.21834 38572751

[2] Morgan E, Soerjomataram I, Rumgay H, Coleman HG, Thrift AP, Vignat J, et al. The global landscape of esophageal squamous cell carcinoma and esophageal adenocarcinoma incidence and mortality in 2020 and projections to 2040: new estimates from GLOBOCAN 2020. Gastroenterology. 2022;163:649–58. 35671803 10.1053/j.gastro.2022.05.054

[3] Yang H, Wang F, Hallemeier CL, Lerut T, Fu J. Oesophageal cancer. Lancet. 2024;404:1991–2005. 39550174 10.1016/S0140-6736(24)02226-8

[4] Ajani JA, D’Amico TA, Bentrem DJ, Cooke D, Corvera C, Das P, et al. Esophageal and esophagogastric junction cancers, version 2.2023, NCCN clinical practice guidelines in oncology. J Natl Compr Canc Netw. 2023;21:393–422. 37015332 10.6004/jnccn.2023.0019

[5] Obermannova R, Alsina M, Cervantes A, Leong T, Lordick F, Nilsson M, et al. Oesophageal cancer: ESMO clinical practice guideline for diagnosis, treatment and follow-up. Ann Oncol. 2022;33:992–1004. 35914638 10.1016/j.annonc.2022.07.003

[6] Kato K, Machida R, Ito Y, Daiko H, Ozawa S, Ogata T, et al. Doublet chemotherapy, triplet chemotherapy, or doublet chemotherapy combined with radiotherapy as neoadjuvant treatment for locally advanced oesophageal cancer (JCOG1109 NExT): A randomised, controlled, open-label, phase 3 trial. Lancet. 2024;404:55–66. 38876133 10.1016/S0140-6736(24)00745-1

[7] Fang K, Sun M, Leng Z, Chu Y, Zhao Z, Li Z, et al. Targeting IGF1R signaling enhances the sensitivity of cisplatin by inhibiting proline and arginine metabolism in oesophageal squamous cell carcinoma under hypoxia. J Exp Clin Cancer Res. 2023;42:73. 36978187 10.1186/s13046-023-02623-2 PMC10044411

[8] Mariette C, Piessen G, Triboulet JP. Therapeutic strategies in oesophageal carcinoma: role of surgery and other modalities. Lancet Oncol. 2007;8:545–53. 17540306 10.1016/S1470-2045(07)70172-9

[9] Rustgi AK, El-Serag HB. Esophageal carcinoma. N Engl J Med. 2014;371:2499–509. 25539106 10.1056/NEJMra1314530

[10] Galluzzi L, Vitale I, Michels J, Brenner C, Szabadkai G, Harel-Bellan A, et al. Systems biology of cisplatin resistance: past, present and future. Cell Death Dis. 2014;5:e1257. 24874729 10.1038/cddis.2013.428 PMC4047912

[11] Zhao Y, Butler EB, Tan M. Targeting cellular metabolism to improve cancer therapeutics. Cell Death Dis. 2013;4:e532. 23470539 10.1038/cddis.2013.60 PMC3613838

[12] Graves JD, Krebs EG. Protein phosphorylation and signal transduction. Pharm Ther. 1999;82:111–21. 10.1016/s0163-7258(98)00056-4 10454190

[13] Manning G, Whyte DB, Martinez R, Hunter T, Sudarsanam S. The protein kinase complement of the human genome. Science. 2002;298:1912–34. 12471243 10.1126/science.1075762

[14] Zhang Q, Yu J, You Q, Wang L. Modulating phosphorylation by proximity-inducing modalities for cancer therapy. J Med Chem. 2024;67:21695–716. 39648992 10.1021/acs.jmedchem.4c02624

[15] Singh V, Ram M, Kumar R, Prasad R, Roy BK, Singh KK. Phosphorylation: implications in cancer. Protein J. 2017;36:1–6. 28108801 10.1007/s10930-017-9696-z

[16] Brushia RJ, Walsh DA. Phosphorylase kinase: the complexity of its regulation is reflected in the complexity of its structure. Front Biosci. 1999;4:D618–41. 10487978 10.2741/brushia

[17] Hanks SK. Homology probing: identification of cDNA clones encoding members of the protein-serine kinase family. Proc Natl Acad Sci USA. 1987;84:388–92. 2948189 10.1073/pnas.84.2.388 PMC304212

[18] Chan KF, Hurst MO, Graves DJ. Phosphorylase kinase specificity. A comparative study with cAMP-dependent protein kinase on synthetic peptides and peptide analogs of glycogen synthase and phosphorylase. J Biol Chem. 1982;257:3655–9. 6277942

[19] Itoh Y, Sanosaka M, Fuchino H, Yahara Y, Kumagai A, Takemoto D, et al. Salt-inducible kinase 3 signaling is important for the gluconeogenic programs in mouse hepatocytes. J Biol Chem. 2015;290:17879–93. 26048985 10.1074/jbc.M115.640821 PMC4505037

[20] Yang WS, Kim KJ, Gaschler MM, Patel M, Shchepinov MS, Stockwell BR. Peroxidation of polyunsaturated fatty acids by lipoxygenases drives ferroptosis. Proc Natl Acad Sci USA. 2016;113:E4966–75. 27506793 10.1073/pnas.1603244113 PMC5003261

[21] Zhu W, Liu D, Lu Y, Sun J, Zhu J, Xing Y, et al. PHKG2 regulates RSL3-induced ferroptosis in Helicobacter pylori-related gastric cancer. Arch Biochem Biophys. 2023;740:109560. 36948350 10.1016/j.abb.2023.109560

[22] Zhang Z, Zhang C, Yang Z, Zhang G, Wu P, Luo Y, et al. M(6)A regulators as predictive biomarkers for chemotherapy benefit and potential therapeutic targets for overcoming chemotherapy resistance in small-cell lung cancer. J Hematol Oncol. 2021;14:190. 34758859 10.1186/s13045-021-01173-4 PMC8579518

[23] Wen D, Xiao H, Gao Y, Zeng H, Deng J. N6-methyladenosine-modified SENP1, identified by IGF2BP3, is a novel molecular marker in acute myeloid leukemia and aggravates progression by activating AKT signal via de-SUMOylating HDAC2. Mol Cancer. 2024;23:116. 38822351 10.1186/s12943-024-02013-y PMC11141000

[24] Lu Y, Zhu J, Zhang Y, Li W, Xiong Y, Fan Y, et al. Lactylation-Driven IGF2BP3-Mediated Serine Metabolism Reprogramming and RNA m6A-Modification Promotes Lenvatinib Resistance in HCC. Adv Sci. 2024;11:e2401399. 10.1002/advs.202401399 PMC11633555 39450426

[25] Zhu Z, Xia X, Lu Y, Li D, He X, Zhang B, et al. PARK7-driven IGF2BP3-K76 lactylation mediates ferroptosis and HAIC resistance in hepatocellular carcinoma. Redox Biol. 2025;87:103869. 41038069 10.1016/j.redox.2025.103869 PMC12514540

[26] Lin Z, Li J, Zhang J, Feng W, Lu J, Ma X, et al. Metabolic reprogramming driven by IGF2BP3 promotes acquired resistance to EGFR inhibitors in Non-Small Cell Lung Cancer. Cancer Res. 2023;83:2187–207. 37061993 10.1158/0008-5472.CAN-22-3059

[27] Wan W, Ao X, Chen Q, Yu Y, Ao L, Xing W, et al. METTL3/IGF2BP3 axis inhibits tumor immune surveillance by upregulating N(6)-methyladenosine modification of PD-L1 mRNA in breast cancer. Mol Cancer. 2022;21:60. 35197058 10.1186/s12943-021-01447-y PMC8864846

[28] Hanniford D, Ulloa-Morales A, Karz A, Berzoti-Coelho MG, Moubarak RS, Sanchez-Sendra B, et al. Epigenetic silencing of CDR1as drives IGF2BP3-Mediated melanoma invasion and metastasis. Cancer Cell. 2020;37:55–70. 31935372 10.1016/j.ccell.2019.12.007 PMC7184928

[29] Pan Z, Zhao R, Li B, Qi Y, Qiu W, Guo Q, et al. EWSR1-induced circNEIL3 promotes glioma progression and exosome-mediated macrophage immunosuppressive polarization via stabilizing IGF2BP3. Mol Cancer. 2022;21:16. 35031058 10.1186/s12943-021-01485-6 PMC8759291

[30] Dai W, Tian R, Yu L, Bian S, Chen Y, Yin B, et al. Overcoming therapeutic resistance in oncolytic herpes virotherapy by targeting IGF2BP3-induced NETosis in malignant glioma. Nat Commun. 2024;15:131. 38167409 10.1038/s41467-023-44576-2 PMC10762148

[31] Huang H, Weng H, Sun W, Qin X, Shi H, Wu H, et al. Recognition of RNA N(6)-methyladenosine by IGF2BP proteins enhances mRNA stability and translation. Nat Cell Biol. 2018;20:285–95. 29476152 10.1038/s41556-018-0045-z PMC5826585

[32] Lin C, He H, Liu H, Li R, Chen Y, Qi Y, et al. Tumour-associated macrophages-derived CXCL8 determines immune evasion through autonomous PD-L1 expression in gastric cancer. Gut. 2019;68:1764–73. 30661053 10.1136/gutjnl-2018-316324

[33] Cambier S, Gouwy M, Proost P. The chemokines CXCL8 and CXCL12: Molecular and functional properties, role in disease and efforts towards pharmacological intervention. Cell Mol Immunol. 2023;20:217–51. 36725964 10.1038/s41423-023-00974-6 PMC9890491

[34] Carpenter ES, Kadiyala P, Elhossiny AM, Kemp SB, Li J, Steele NG, et al. KRT17high/CXCL8+ tumor cells display both classical and basal features and regulate myeloid infiltration in the pancreatic cancer microenvironment. Clin Cancer Res. 2024;30:2497–513. 37851080 10.1158/1078-0432.CCR-23-1421 PMC11024060

[35] Inoue M, Takeuchi H, Matsuda S, Nishi T, Fukuda K, Nakamura R, et al. IL-8/CXCR2 signalling promotes cell proliferation in oesophageal squamous cell carcinoma and correlates with poor prognosis. Anticancer Res. 2021;41:783–94. 33517283 10.21873/anticanres.14830

[36] Patel A, Lee HO, Jawerth L, Maharana S, Jahnel M, Hein MY, et al. A Liquid-to-Solid phase transition of the ALS protein FUS accelerated by disease mutation. Cell. 2015;162:1066–77. 26317470 10.1016/j.cell.2015.07.047

[37] Mann JR, Gleixner AM, Mauna JC, Gomes E, DeChellis-Marks MR, Needham PG, et al. RNA binding antagonizes neurotoxic phase transitions of TDP-43. Neuron. 2019;102:321–38. 30826182 10.1016/j.neuron.2019.01.048 PMC6472983

[38] Monahan Z, Ryan VH, Janke AM, Burke KA, Rhoads SN, Zerze GH, et al. Phosphorylation of the FUS low-complexity domain disrupts phase separation, aggregation, and toxicity. EMBO J. 2017;36:2951–67. 28790177 10.15252/embj.201696394 PMC5641905

[39] McGurk L, Gomes E, Guo L, Mojsilovic-Petrovic J, Tran V, Kalb RG, et al. Poly(ADP-Ribose) prevents pathological phase separation of TDP-43 by promoting liquid demixing and stress granule localization. Mol Cell. 2018;71:703–17. 30100264 10.1016/j.molcel.2018.07.002 PMC6128762

[40] Cheng Y, Xie W, Pickering BF, Chu KL, Savino AM, Yang X, et al. N(6)-Methyladenosine on mRNA facilitates a phase-separated nuclear body that suppresses myeloid leukemic differentiation. Cancer Cell. 2021;39:958–72. 34048709 10.1016/j.ccell.2021.04.017 PMC8282764

[41] Guillen-Boixet J, Kopach A, Holehouse AS, Wittmann S, Jahnel M, Schlussler R, et al. RNA-induced conformational switching and clustering of G3BP drive stress granule assembly by condensation. Cell. 2020;181:346–61. 32302572 10.1016/j.cell.2020.03.049 PMC7181197

[42] Liang Q, Peng N, Xie Y, Kumar N, Gao W, Miao Y. MolPhase, an advanced prediction algorithm for protein phase separation. EMBO J. 2024;43:1898–918. 38565952 10.1038/s44318-024-00090-9 PMC11065880

[43] Nakamura T, Hipp C, Santos Dias Mourao A, Borggrafe J, Aldrovandi M, Henkelmann B, et al. Phase separation of FSP1 promotes ferroptosis. Nature. 2023;619:371–7. 37380771 10.1038/s41586-023-06255-6 PMC10338336

[44] Piovesan D, Del Conte A, Mehdiabadi M, Aspromonte MC, Blum M, Tesei G, et al. MOBIDB in 2025: integrating ensemble properties and function annotations for intrinsically disordered proteins. Nucleic Acids Res. 2025;53:D495–503. 39470701 10.1093/nar/gkae969 PMC11701742

[45] Gu X, Zhu Y, Su J, Wang S, Su X, Ding X, et al. Lactate-induced activation of tumor-associated fibroblasts and IL-8-mediated macrophage recruitment promote lung cancer progression. Redox Biol. 2024;74:103209. 38861833 10.1016/j.redox.2024.103209 PMC11215341

[46] Giudice E, Huang T, Nair JR, Zurcher G, McCoy A, Nousome D, et al. The CHK1 inhibitor prexasertib in BRCA wild-type platinum-resistant recurrent high-grade serous ovarian carcinoma: a phase 2 trial. Nat Commun. 2024;15:2805. 38555285 10.1038/s41467-024-47215-6 PMC10981752

[47] Gupta N, Huang T, Nair JR, An D, Zurcher G, Lampert EJ, et al. BLM overexpression as a predictive biomarker for CHK1 inhibitor response in PARP inhibitor-resistant BRCA-mutant ovarian cancer. Sci Transl Med. 2023;15:d7872. 10.1126/scitranslmed.add7872 PMC10758289 37343085

[48] Do KT, Kochupurakkal B, Kelland S, de Jonge A, Hedglin J, Powers A, et al. Phase 1 combination study of the CHK1 inhibitor prexasertib and the PARP inhibitor olaparib in high-grade serous ovarian cancer and other solid tumors. Clin Cancer Res. 2021;27:4710–6. 34131002 10.1158/1078-0432.CCR-21-1279

[49] Chen Y, Ye J, Zhu Z, Zhao W, Zhou J, Wu C, et al. Comparing paclitaxel plus fluorouracil versus cisplatin plus fluorouracil in chemoradiotherapy for locally advanced esophageal squamous cell cancer: a randomized, multicenter, phase III clinical trial. J Clin Oncol. 2019;37:1695–703. 30920880 10.1200/JCO.18.02122 PMC6638596

[50] Wong I, Lam KO, Zhang RQ, Chan W, Wong C, Chan F, et al. Neoadjuvant chemoradiotherapy using cisplatin and 5-Fluorouracil (PF) versus carboplatin and paclitaxel (CROSS regimen) for esophageal squamous cell carcinoma (ESCC): a propensity score-matched study. Ann Surg. 2020;272:779–85. 32833766 10.1097/SLA.0000000000004329

[51] Chen E, He Y, Jiang J, Yi J, Zou Z, Song Q, et al. CDCA8 induced by NF-YA promotes hepatocellular carcinoma progression by regulating the MEK/ERK pathway. Exp Hematol Oncol. 2023;12:9. 36639822 10.1186/s40164-022-00366-y PMC9838039

[52] Yu ZY, Jiang XY, Zhao RR, Qin JJ, Luo CJ, Ren YX, et al. Effect of KIF22 on promoting proliferation and migration of gastric cancer cells via MAPK-ERK pathways. Chin Med J. 2020;133:919–28. 32187050 10.1097/CM9.0000000000000742 PMC7176455

[53] Chen J, Zhang Z, Ni J, Sun J, Ju F, Wang Z, et al. ENO3 promotes colorectal cancer progression by enhancing cell glycolysis. Med Oncol. 2022;39:80. 35477821 10.1007/s12032-022-01676-1

[54] Ling A, Lofgren-Burstrom A, Larsson P, Li X, Wikberg ML, Oberg A, et al. TAP1 down-regulation elicits immune escape and poor prognosis in colorectal cancer. Oncoimmunology. 2017;6:e1356143. 29147604 10.1080/2162402X.2017.1356143 PMC5674960

[55] Lin YC, Liu D, Chakraborty A, Kadyrova LY, Song YJ, Hao Q, et al. Orc6 is a component of the replication fork and enables efficient mismatch repair. Proc Natl Acad Sci USA. 2022;119:e2121406119. 35622890 10.1073/pnas.2121406119 PMC9295755

[56] Eun JW, Yoon JH, Ahn HR, Kim S, Kim YB, Lim SB, et al. Cancer-associated fibroblast-derived secreted phosphoprotein 1 contributes to resistance of hepatocellular carcinoma to sorafenib and lenvatinib. Cancer Commun. 2023;43:455–79. 10.1002/cac2.12414 PMC10091107 36919193

[57] Shi CJ, Pang FX, Lei YH, Deng LQ, Pan FZ, Liang ZQ, et al. 5-Methylcytosine methylation of MALAT1 promotes resistance to sorafenib in hepatocellular carcinoma through ELAVL1/SLC7A11-mediated ferroptosis. Drug Resist Updat. 2025;78:101181. 39657434 10.1016/j.drup.2024.101181

[58] Ke W, Zhang L, Zhao X, Lu Z. P53 m(6)A modulation sensitizes hepatocellular carcinoma to apatinib through apoptosis. Apoptosis. 2022;27:426–40. 35503144 10.1007/s10495-022-01728-x

[59] Yang S, Zhou X, Jia Z, Zhang M, Yuan M, Zhou Y, et al. Epigenetic regulatory mechanism of ADAMTS12 expression in osteoarthritis. Mol Med. 2023;29:86. 37400752 10.1186/s10020-023-00661-2 PMC10318776

[60] Zhang X, Sun J, Zheng X, Zhang J, Tan L, Fan L, et al. Plin4 exacerbates cadmium-decreased testosterone levels via inducing ferroptosis in testicular Leydig cells. Redox Biol. 2024;76:103312. 39173539 10.1016/j.redox.2024.103312 PMC11387904

[61] Dong F, Qin X, Wang B, Li Q, Hu J, Cheng X, et al. ALKBH5 facilitates Hypoxia-Induced paraspeckle assembly and IL8 secretion to generate an immunosuppressive tumor microenvironment. Cancer Res. 2021;81:5876–88. 34670781 10.1158/0008-5472.CAN-21-1456

