# Supplementary material for: PHKG2 confers resistance of ESCC to cisplatin and enhances CXCL8-dependent immunosuppression to exacerbate tumorigenesis
Source: Cell Death Dis. 2026 May 20;17(1):723. doi: 10.1038/s41419-026-08721-1 (PMC13473487; doi:10.1038/s41419-026-08721-1)
Supplement: Supplementary file 1 [file 41419_2026_8721_MOESM1_ESM.docx]

**PHKG2 confers resistance of ESCC to cisplatin and enhances CXCL8-dependent immunosuppression to exacerbate tumorigenesis** Jiayan Wu^1,2Ψ^, Chengrui Zhong^2,3Ψ^, Haosheng Zheng^1,2Ψ^, Fei Qin^1,2^, Xianyu Qin^1,2^, Yuzhen Zheng^1,2^, Junguo Chen^1,2^, Gengfeng Wang^1,2^, Bozhu Jian^1,2^, Zui Liu^1,2^, Xingping Yang^4^, Chao Cheng^5,6,7^, Guolin Li^2,3^*，Hongying Liao^1,2^*

1.Department of Thoracic Surgery, The Sixth Affiliated Hospital, Sun Yat-sen University, Guangzhou, China.

2.Biomedical Innovation Center, The Sixth Affiliated Hospital, Sun Yat-sen University, Guangzhou, China.

3.Department of General Surgery (Hepatobiliary, Pancreatic and Splenic Surgery), The Sixth Affiliated Hospital, Sun Yat-sen University,Guangzhou, China.

4.University of Burgundy, Dijon, France.

5.Institute for Clinical and Translational Research, Baylor College of Medicine, Houston, TX, USA.

6.Section of Epidemiology and Population Sciences, Department of Medicine, Baylor College of Medicine, Houston, TX, USA.

7.Dan L Duncan Comprehensive Cancer Center, Baylor College of Medicine, Houston, TX, USA.

^Ψ^ These authors contributed equally to this work

*Correspondence to: Hongying Liao, Department of Thoracic Surgery, The Sixth Affiliated Hospital,Sun Yat-sen University, No.26 ErHeng Road, Guangzhou 510655, China. Email: liaohy2@mail.sysu.edu.cn,+8602035919121；Guolin Li, Department of Hepatobiliary, Pancreatic and Splenic Surgery, The Sixth Affiliated Hosptial, Sun Yat-sen University, No.26 ErHeng Road, Guangzhou 510655, China. Email: [liglin@mail.sysu.edu.cn](mailto:liglin@mail.sysu.edu.cn;),+8602038349697

**Additional Supplementary Files**

**File Name: Supplementary Data 1**

Description: All sgRNA sequence of CRISPR-Cas9 knockout library screen.

**File Name: Supplementary Data 2**

Description:Genome-scale CRISPR-Cas9 knockout library screening identified all differentially down-regulated genes.

**File Name: Supplementary Data 3**

Description: Transcriptome sequencing of the parental TE-1 line and the DDP-resistant subline TE-1R identified all differentially upregulated genes.

**File Name: Supplementary Data 4**

Description: Transcriptome sequencing of clinical specimens from patients responsive and non-responsive to DDP-based chemotherapy identified all differentially upregulated genes.

**File Name: Supplementary Data 5**

Description: Proteins in KYSE-150 cell lysates were immunoprecipitated (IP) and then analyzed by mass spectrometry.

**File Name: Supplementary Data 6**

Description: Proteins from TE-1 cell lysates were immunoprecipitated (IP) and subsequently analyzed via mass spectrometry.

**File Name: Supplementary Data 7**

Description: Protein modifications in TE-1 cell lysates were detected by immunoprecipitation (IP) and then analyzed using mass spectrometry.

**File Name: Supplementary Data 8**

Description: MeRIP (m6A)-sequencing in TE-1 cells identified m6A-modified transcripts.

**File Name: Supplementary Data 9**

Description: Transcriptomic analysis revealed significantly differentially expressed genes in PHKG2-overexpressing cells compared to control cells.

**File Name: Supplementary Data 10**

Description: Transcriptomic analysis revealed significantly differentially expressed genes in IGF2BP3-overexpressing cells compared to control cells.

**File Name: Supplementary Data 11**

Description: The top 200 hits from virtual screening of a chemical library comprising 306,100 small-molecule drugs.

**File Name: Supplementary Data 12**

Description: Supplementary Figures and Tables.

**Supplementary materials and methods**

**Proximity ligation assay (PLA)**

According to the manufacturer’s protocol, Proximity Ligation Assays (PLA) were performed using the Duolink PLA kit (Sigma). The following methodology was followed to conduct PLAs using a Duolink kit with the included chemicals and buffers (DUO92101; Sigma-Aldrich). Cells were plated out on glass coverslips in a 24-well plate. Cells were fixed with cold 4% paraformaldehyde (PFA) for 15 minutes at RT after being washed three times with PBS. After fixing the cells in 4% PFA, they were washed in PBS, permeabilized with 0.1% Triton X-100 in PBS for 15 minutes at room temperature, incubated in blocking buffer (supplied in the kit) for 1 hour at 37 °C in a humidified chamber, and then incubated with various primary antibodies in antibody diluents overnight at 4 °C. After being incubated with the PLA probes for 1 hour at 37 °C in a humidified environment, cells were washed three times for a total of 15 minutes in buffer A. Next, cells were incubated for 1 hour with the ligation reaction mix at 37 degrees Celsius, followed by a 3 × 5-minute wash in buffer A. Finally, the reaction was incubated with the amplification mix at 37 degrees Celsius in a dark humid chamber for 140 minutes. Samples were washed with 2x buffer B for ten minutes, then with 0.01x buffer for one minute, before being processed with mounting media. We used a Zeiss LSM880 confocal microscope to take pictures of DAPI-stained cells.

**In vitro kinase assay**

The pcDNA3-HA-PHKG2 were transfected into HEK293T cells by using lipofectamine 3000 (ThermoFisherScientific). Three days after transfection, cells were lysed with lysis buffer (20 mM Tris-HCl (pH 8.0), 0.5% Nonidet P-40, 250 mM NaCl, 3 mM EDTA, 3 mM EGTA, 2 mM DTT, 0.5 mM phenylmethylsulfonyfluoride (PMSF), 20 mM beta-glycerolphosphate, 1 mM sodium orthovanadate and 1 mg/ml leupeptin. HA-PHKG2 proteins were immunoprecipitated by using a mouse anti-HA.11 epitope Tag antibody (BioLegend, Cat # 901516) and protein G-agarose beads (Sigma-Aldrich) by rotation at 4 C overnight. The precipitated PHKG2 proteins were re-suspended in 40 ml of 1x kinase buffer (Cell Signaling, Cat #9802) supplemented with 200 mM ATP (Cell Signaling) and purified His-IGF2BP3 WT, S11A, T21A or 2A protein, which was expressed in pET-28a vector in E. coli. The kinase reaction was conducted at 30 C for 30 min, and was stopped by addition of 20 ml 3x SDS sample buffer. Each sample was then boiled for 10 min at 100 C, followed by IB analysis with indicated antibodies.

**Surface Plasmon Resonance (SPR)**

Interactions between the recombinant PHKG2 protein and prexasertib were analyzed using the Biacore T200 system. Briefly, recombinant PHKG2 protein was immobilized on a sensor chip, using an amine coupling kit (GE Healthcare). The final PHKG2-immobilized level was typically 1 mg/mL. Subsequently, prexasertib was injected as analysis at various concentrations and using DMSO as running buffer. For binding studies, analysis was applied at corresponding concentrations in running buffer at a flow rate of 30 mL/min. Chip platforms were washed with running buffer and 50% DMSO. IA Evaluation Software was used for data analysis.

**Supplementary Fig. 1 CRISPR screening and transcriptomic profiling of DDP-resistant cells and patient samples identify PHKG2 as a candidate driver of DDP resistance in ESCC.**

**A** The IC50 of DDP in KYSE-150, TE-1 and Eca109 cells(n=3). **B** Sequencing quality metrics: total reads and mapping efficiency. **C** sgRNA sequencing depth distribution. **D** CRISPR library uniformity assessment. **E** Volcano plot of genome-wide CRISPR screen identifies differential sgRNAs under cisplatin treatment. **F** The IC50 of DDP in TE-1 parental and TE-1-R cells, KYSE-150 and KYSE-150-R(n=3). **G** RNA-seq volcano plot: differentially expressed genes in cisplatin-resistant ESCC cells(n=3). **H** RNA-seq volcano plot: transcriptional differences between non-responders and responders to cisplatin-based therapy. **I** Dose-responsive PHKG2 mRNA upregulation in TE-1 cells (cisplatin: 0, 10, 30 μM; 48 h). **J** Dose-dependent PHKG2 protein elevation in TE-1 cells (cisplatin: 0, 10, 30 μM; 48 h). **K** Reduced cisplatin-induced apoptosis in TE-1-R versus parental cells (30 μM, Annexin V flow cytometry, n=3). **L** Representative images of ESCC patients with low vs. high PHKG2 expression determined by IHC. **M** Kaplan-Meier survival curves were plotted to correlate PHKG2 expression with DFS in ESCC. **N** Pre-and post-treatment computed tomography images of ESCC patients with low vs. high PHKG2 expression receiving DDP-based neoadjuvant therapy.

**Supplementary Fig. 2 PHKG2 drives DDP resistance in vitro and in vivo.**

**A** qPCR analysis of PHKG2 mRNA levels in PHKG2 KO versus control TE-1 cells(n=3). **B** Western blot analysis of PHKG2 protein in PHKG2-KO TE-1 cells. **C** PHKG2-KO effects on TE-1 cell proliferation under 1μM DDP (CCK-8 assay, n=3). **D** Colony formation assay assessing PHKG2-KO-mediated growth with/without 1μM DDP(n=3). **E** PHKG2-KO enhances DDP-induced apoptosis(30 μM, Annexin V flow cytometry, n=3). **F** Apoptosis sensitization by PHKG2 knockout/knockdown (30 μM DDP, flow cytometry, n=3). **G** Schematic of the ESCC xenograft model treated with DDP(5 mg/kg). **H** IHC analysis of Ki67 and cleaved caspase 3 in TE-1 xenografts treated with or without DDP (5 mg/kg).

**Supplementary Fig. 3PHKG2 stabilizes IGF2BP3 protein through phosphorylation of IGF3BP3 at T225 and T306.**

**A-B** Co-immunoprecipitation followed by western blot analysis confirmed the interaction between endogenous PHKG2 and IGF2BP3 in TE-1 and TE-1-R cells. **C** Immunofluorescence staining demonstrated cytoplasmic co-localization of PHKG2 (green) and IGF2BP3 (red) in esophageal squamous cell carcinoma patient tissue samples. **D-E** IGF2BP3 mRNA and protein expression levels in PHKG2-overexpressing Eca109 cells were compared with those in control cells(n=3). **F-I** IGF2BP3 mRNA and protein levels were analyzed in PHKG2-knockout/knockdown KYSE-150, TE-1, and TE-1-R cells relative to control groups(n=3). **J** LC-MS/MS analysis characterized the fragmentation spectrum of the IGF2BP3 T306 peptide. **K** Western blot analysis of immunoprecipitated HA-tagged IGF2BP3 wild-type (WT), T225A, T306A, and T225A/T306A (2A) mutants in 293T cells showed dramatically reduced phosphorylation in the 2A mutant. **L** Western blot analysis assessed IGF2BP3 protein stability in KYSE-150 and TE-1 cells with or without proteasome inhibitor MG132 treatment. **M** Western blotting was used to determine whether MG132 treatment rescued IGF2BP3 protein levels in PHKG2-knockdown TE-1 cells.

**Supplementary Fig. 4 PHKG2 promotes DDP resistance through IGF2BP3 in vitro and in vivo.**

**A** Apoptosis induced by IGF2BP3 knockdown was analyzed by flow cytometry with Annexin V staining in PHKG2-overexpressing Eca109 cells treated with 30 μM cisplatin(n=3). **B** Cell proliferation following IGF2BP3 knockdown in KYSE-150-R cells was measured using the Cell Counting Kit-8(CCK-8) assay(n=3). **C** Cell proliferation following IGF2BP3 knockdown was evaluated via clone formation assay in TE-1-R and KYSE-150-R cells(n=3). **D** Flow cytometry with Annexin V staining was used to assess apoptosis in TE-1-R and KYSE-150-R cells treated with 30 μM DDP following IGF2BP3 knockdown(n=3). **E** Clone formation assays were performed to determine the effect of IGF2BP3 overexpression on cell proliferation in PHKG2-knockout KYSE-150 cells(n=3). **F** Cell proliferation was measured in PHKG2-knockdown TE-1-R cells after overexpression of IGF2BP3 wild-type (WT), T225A, or T306A mutants(n=3). **G** Apoptosis was assessed by flow cytometry in IGF2BP3-knockdown TE-1-R cells and control cells treated with 30 μM DDP, following overexpression of IGF2BP3-WT or T225A/T306A mutants(n=3). **H** Flow cytometry analysis of apoptosis was conducted in PHKG2-knockout Eca109 cells with or without PHKG2 overexpression, following introduction of IGF2BP3-WT or T225A/T306A mutants and treatment with DDP(n=3).

**Supplementary Fig. 5 PHKG2 and IGF2BP3 regulate CXCL8 expression in an m6A-dependent manner.**

**A-B** Distribution of N6-methyladenosine (m6A) modifications in messenger RNA (mRNA) and long non-coding RNA (lncRNA) transcripts. The m6A signal was predominantly enriched in coding sequence (CDS) regions of mRNAs. **C** Top 10 gene ontology (GO) biological process terms for genes with m6A modifications identified by MeRIP-m6A-seq. **D** Volcano plot depicting differentially expressed genes identified by RNA sequencing (RNA-seq) analysis comparing IGF2BP3-overexpressing TE-1 cells with control cells. **E** Volcano plot showing differentially expressed genes from RNA-seq analysis of PHKG2-overexpressing TE-1 cells versus control cells. **F-G** q-PCR analysis of CXCL8 mRNA expression in PHKG2-knockout, IGF2BP3-knockdown, and control KYSE-150 and TE-1 cells$n=3$. **G-I** Western blot analysis of CXCL8 protein levels in PHKG2-KO, IGF2BP3-KD, and control KYSE-150 and TE-1 cells. **J-K** CXCL8 mRNA and protein expression in PHKG2-OE Eca109 cells and control cells, assessed by qPCR and Western blotting, respectively(n=3). **L** CXCL8 protein expression in control, PHKG2-KD, and PHKG2-KD + IGF2BP3-OE KYSE-150 cells. **M-N** Impact of IGF2BP3-KD and PHKG2-KO on CXCL8 mRNA stability, evaluated via actinomycin D chase assays in KYSE-150 and TE-1 cells(n=3). **O** Dot blot analysis of global m6A levels in KYSE-150 cells treated with 50 μM 3-deazaadenosine (DAA) or DMSO vehicle control, with methylene blue (MB) staining as a loading control. **P** Western blot analysis of CXCL8 protein expression in KYSE-150 cells treated with 50 μM DAA or vehicle control. **Q** qPCR was performed to detect the expression of five m⁶A writers in ESCC cells(n=3). **R** q-PCR analysis of WTAP mRNA levels in WTAP KD and control ESCC cells(n=3). **S** qPCR was performed to detect the expression of CXCL8 mRNA in ESCC cells after WTAP KD(n=3). **T** mRNA degradation rate of CXCL8 in WTAP KD TE-1R cells treated with actinomycin D(n=3).

**Supplementary Fig. 6 The effect of PHKG2 and IGF2BP3 on DDP resistance is dependent**

**on CXCL8 expression**

**A** Western blot analysis confirmed efficient knockdown of CXCL8 using two independent shRNAs. **B** Western blot analysis verified successful overexpression of CXCL8 in PHKG2-knockdown TE-1 cells. **C** Colony formation assays evaluated the effect of CXCL8 overexpression on the proliferation of PHKG2-knockdown TE-1 cells treated with 1 μM cisplatin(n = 3). **D-E** CXCL8 expression levels in CXCL8-knockdown TE-1-R cells and control cells were analyzed by qPCR and Western blot, respectively(n = 3). **F-G** CCK-8 assays assessed the impact of CXCL8 knockdown on TE-1-R cell growth under both 1 μM DDP treatment and untreated conditions. **H** Colony formation assays determined the effects of CXCL8 knockdown on TE-1-R cell proliferation with and without 1 μM DDP exposure(n = 3). **I** Flow cytometry analysis of Annexin V staining evaluated the effect of CXCL8 knockdown on DDP (30 μM)-induced apoptosis in KYSE-150 and TE-1 cells(n = 3). **J-L** Immunohistochemical (IHC) staining for Ki67 and cleaved caspase 3 was performed on subcutaneous tumor tissues from Eca109 cells, PHKG2-overexpressing Eca109 cells, and PHKG2-overexpressing/CXCL8-knockdown Eca109 cells treated with DDP or vehicle control(n = 6).

**Supplementary Fig. 7 Clinical significance of targeting PHKG2 in ESCC.**

**A** Tumor weights of ESCC PDX models at the endpoint of the experiment treated with siRNA(n = 3). **B** Representative immunohistochemical images and quantitative analysis of Ki67 expression in ESCC PDX tumor tissues treated with siRNA(n = 3). **C** Representative immunohistochemical images and quantitative analysis of cleaved caspase 3 expression in ESCC PDX tumor tissues treated with siRNA(n = 3). **D** 2D diagrams of prexasertib and PHKG2 protein molecules. **E** Tumor growth curves of ESCC PDX models across experimental groups(n=3). **F** Representative immunohistochemical images of Ki67 and cleaved caspase 3 expression in ESCC PDX tumor tissues treated with or without prexasertib. **G-H** Quantitative analysis of Ki67 and cleaved caspase 3 expression in ESCC PDX tumor tissues treated with or without prexasertib(n = 6). **I** Metabolite quantitative analysis of the shPHKG group compared with the NC group under DDP( (n = 3).

**Supplementary Fig. 8 The gating strategy for the Annexin V-FICT/PI apoptosis assay, measured via flow cytometry.**

| **Supplementary Table 1.Antibodies included in the study.** | | |
| --- | --- | --- |
| **Antibodies** | **IDENTIFIER** | **Catalogue Number** |
| Anti-GAPDH | Proteintech | 60004-1-Ig |
| Anti-PHKG2 | Proteintech | 15109-1-AP |
| Anti-IGF2BP3 | Proteintech | 14642-1-AP |
| Anti-CXCL8 | Proteintech | 17038-1-AP |
| Anti-His | Proteintech | 66005-1-lg |
| Anti-HA | Proteintech | 51064-2-AP |
| Anti-mouse  IgG, HRP-linked Antibody | Cell Signaling Technology | #7076S |
| Anti-rabbit IgG, HRP-linked Antibody | Cell Signaling Technology | #7074S |
| Anti-PHKG2 | ThemoFisher Scientific | # MA5-19594 |
| Alexa Fluor 488-conjugated Goat Anti-Rabbit IgG (H+L) | Servicebio | GB25303 |
| Alexa Fluor® 594-conjugated Goat Anti-Mouse IgG (H+L) | Servicebio | GB28303 |
| Anti-m6A | Synaptic Systems | 202003 |
| Anti-Ki67 | ab279653 | Abcam |
| Anti-Cleaved Caspase-3 | Cell Signaling Technology | #9664 |

| **Supplementary Table 2. The primers used in present study** | | |
| --- | --- | --- |
| **Primer**  **names** | **Forward (5’-3’)** | **Reverse (5’-3’)** |
| PHKG2 | GAGTTTTACCAGAAGTACGACCC | GTAGCTCGATGAACACAACG |
| IGF2BP3 | TCGTGACCAGACACCTGATGAG | GGTGCTGCTTTACCTGAGTCAG |
| CXCL8 | GAGAGTGATTGAGAGTGGACCAC | CACAACCCTCTGCACCCAGTTT |
| GAPDH | TCCAAAATCAAGTGGGGCGA | AAATGAGCCCCAGCCTTCTC |

| **Supplementary Table 3. Sequences of RNA Oligonucleotides** | | |
| --- | --- | --- |
| **Name** | **Sense strand/sense primer (5’-3’)** | **Antisense strand/antisense primer**  **(5’-3’)** |
| **siRNA** | | |
| siPHKG2-1 | GCCCGAGAAUAUUCUCCUATT | UAGGAGAAUAUUCUCGGGCTT |
| siPHKG2-1 | UCACCCUCAUCGAUUCCUATT | UAGGAAUCGAUGAGGGUGATT |
| siIGF2BP3-1 | CCUUGAAAGUAGCCUAUAUTT | AUAUAGGCUACUUUCAAGGTT |
| siIGF2BP3-2 | GCAGGAAUUGACGCUGUAUTT | AUACAGCGUCAAUUCCUGCTT |
| **shRNA** | | |
| shCXCL8-1 | GCUCUCUUGGCAGCCUUCCUGAUUU | AAAUCAGGAAGGCUGCCAAGAGAGC |
| shCXCL8-2 | GAACUUAGAUGUCAGUGCAUAAAGA | UCUUUAUGCACUGACAUCUAAGUUC |
| **sgRNA** | | |
| sgPHKG2-1 | ATCTCGATGCACAATGTTGT |  |
| sgPHKG2-2 | ACCAGTTCAGTTCCCCCGAG |  |
