# Supplementary material for: PHKG2 confers resistance of ESCC to cisplatin and enhances CXCL8-dependent immunosuppression to exacerbate tumorigenesis
Source: Cell Death Dis. 2026 May 20;17(1):723. doi: 10.1038/s41419-026-08721-1 (PMC13473487; doi:10.1038/s41419-026-08721-1)
Supplement: Supplementary file 2 [file 41419_2026_8721_MOESM2_ESM.docx]

uncropped blots Fig. 1&S1

uncropped blots Fig. 2&S2

uncropped blots Fig. 3&S3

uncropped blots Fig. 4

uncropped blots Fig. 5&S5

uncropped blots Fig. S6

uncropped blots Fig. 9
